# Long-Term Dynamics of Three Dimensional Telomere Profiles in Circulating Tumor Cells in High-Risk Prostate Cancer Patients Undergoing Androgen-Deprivation and Radiation Therapy

**DOI:** 10.3390/cancers11081165

**Published:** 2019-08-14

**Authors:** Landon Wark, Harvey Quon, Aldrich Ong, Darrel Drachenberg, Aline Rangel-Pozzo, Sabine Mai

**Affiliations:** 1 Cell Biology, Research Institute of Oncology and Hematology, University of Manitoba, CancerCare Manitoba, Winnipeg, MB R3E 0V9, Canada; 2 Manitoba Prostate Center, Cancer Care Manitoba, Section of Urology, Department of Surgery, University of Manitoba, Winnipeg, MB R3E 0V9, Canada

**Keywords:** androgen deprivation therapy, radiotherapy, localized high-risk prostate cancer, circulating tumor cells, three-dimensional (3D) telomere profiling

## Abstract

Patient-specific assessment, disease monitoring, and the development of an accurate early surrogate of the therapeutic efficacy of locally advanced prostate cancer still remain a clinical challenge. Contrary to prostate biopsies, circulating tumor cell (CTC) collection from blood is a less-invasive method and has potential as a real-time liquid biopsy and as a surrogate marker for treatment efficacy. In this study, we used size-based filtration to isolate CTCs from the blood of 100 prostate cancer patients with high-risk localized disease. CTCs from five time points: +0, +2, +6, +12 and +24 months were analyzed. Consenting treatment-naïve patients with cT3, Gleason 8-10, or prostate-specific antigen > 20 ng/mL and non-metastatic prostate cancer were included. For all time points, we performed 3D telomere-specific quantitative fluorescence in situ hybridization on a minimum of thirty isolated CTCs. The patients were divided into five groups based on the changes of number of telomeres vs. telomere lengths over time and into three clusters based on all telomere parameters found on diagnosis. Group 2 was classified as non-respondent to treatment and the Cluster 3 presented more aggressive phenotype. Additionally, we compared our telomere results with the PSA levels for each patient at 6 months of ADT, at 6 months of completed RT, and at 36 months post-initial therapy. CTCs of patients with PSA levels above or equal to 0.1 ng/mL presented significant increases of nuclear volume, number of telomeres, and telomere aggregates. The 3D telomere analysis of CTCs identified disease heterogeneity among a clinically homogeneous group of patients, which suggests differences in therapeutic responses. Our finding suggests a new opportunity for better treatment monitoring of patients with localized high-risk prostate cancer.

## 1. Introduction

Androgen deprivation therapy (ADT) combined with radiotherapy (RT) is a standard treatment for patients with localized high-risk prostate cancer (PCa) [1]. However, many patients will eventually regress after therapy and develop metastatic disease [2]. Therefore, there is an urgent need for a biomarker, which can reliably identify patients at high risk for recurrence and metastases [2]. For those patients, intensification of treatment beyond standard combination of ADT with radiation may be required [2].

All the options of intensification of therapy, including longer durations of ADT, combination with intense androgen blockade, chemotherapy or novel agents to target androgen activity through different pathways [3], are associated with additional toxicity. Therefore, it is critically important to ascertain the subgroup of patients in need of that next step. While clinical factors such as prostate-specific antigen (PSA) values, T-category, and Gleason scores have traditionally been used to risk-stratify prostate cancer patients, their accuracy is low (25%–40%) to predict important end points such as progression or metastases after radiation and ADT [4,5]. Due to the lack of a better surrogate, PSA measurement continues to be the main method to monitor treatment response and recurrence after treatment for prostate cancer [6]. However, this is fraught with difficulty as there is no reliable method to differentiate PSA produced by tumor vs. normal prostate tissue [5].

Many studies have evaluated the use of circulating tumor cells (CTCs) as a biomarker to predict disease progression and survival in patients with metastatic, advanced, or even early-stage PCa, as well as an endpoint marker in clinical trials [7,8,9,10,11,12]. As CTCs are responsible for distant metastasis, their analysis could potentially provide information about treatment response [7]. High CTC numbers are associated with aggressive disease, increased metastasis, and decreased time to relapse in men with castration-resistant and metastatic prostate cancer [10,12,13,14]. However, the value of CTCs detection in men with localized high-risk prostate cancer is unknown. Even though CTC collection from blood is a less-invasive method and can be used as a real-time liquid biopsy during regular follow-up [7], there are many challenges for the use of CTCs as a prognostic and/or predictive biomarker. For example, the number of CTCs found in patient samples depends on the isolation method used, because of CTCs immunophenotype heterogeneity, CTCs derived from the same tumor can present different expression of epithelial markers, such as EpCAM [15]. This difference might limit CTC detection by EpCAM-dependent technologies, like CellSearch [15]. In addition, many apoptotic CTC cells are also isolated and analyzed which may not necessarily be representative of potential metastatic cells; and, finally, CTCs can be absent in some non-metastatic PCa patients [15,16]. For a better clinical use of CTCs in PCa as a prognostic and/or predictive biomarker, a combination of enrichment (isolation), detection (identification), and characterization strategies (such as molecular profile), are necessary to improve our ability to identify high-risk lethal prostate cancer in patients with clinically localized high-risk prostate cancers [17,18]. 

In previous studies, we have demonstrated the potential of single-cell analysis of CTCs, combining a filtration-based CTC isolation technology with prostate cancer cell-specific antibodies, followed by the use of 3D telomere profiling to identify PCa patient subgroups [18,19]. Telomere shortening is one of the earliest molecular genomic events in prostate cancer tumorigenesis and can generate genomic instability [20]. The detection of shorter telomeres is associated with increased occurrence of lethal prostate cancer and decreased survival [21]. Additionally, androgen receptor (AR) inactivation by knockdown, androgen deprivation, or treatment with bicalutamide in LNCaP cells (prostate cancer cell line) can induce telomere breaks and telomere fusion [22]. However, telomere dysfunction was not observed following bicalutamide treatment in the AR-negative PC-3 prostate cell line [23]. Clinical studies assessing the effects of ADT on telomeres using prostate cancer patient samples are limited. In 2017, Cheung et al. reported no evidence that ADT deprivation accelerates telomerase shortening in men who have been diagnosed with prostate cancer [24]. However, leucocyte DNA was used for this analysis [24].

In a previous work, we began to follow the early dynamics of CTCs using 3D telomere analysis during ADT (a pilot study composed of 20 patients, where consenting treatment-naïve patients with cT3, Gleason 8-10, or prostate-specific antigen > 20 ng/mL and non-metastatic prostate cancer were included) [19]. We analyzed CTCs from high-risk prostate cancer patient´s samples at different time points: before ADT and RT (+0 month, untreated), after 2 months of ADT (+2 months) but prior RT, and 2 months after the final fraction of RT (+6 months). ADT begun 2 months before the start of RT and continued after RT was completed. At each time point, we enumerated CTCs from the blood, collected PSA values and investigated the nuclear 3D telomere architecture in CTCs derived from patients with non-metastatic high-risk prostate cancer before, post-ADT, and post-RT [19]. Contrary to CTC enumeration and PSA serum levels, we showed that nuclear 3D telomere architectural analysis is highly sensitive in detecting cellular events that affect the genome stability in CTCs, and we described three distinct telomere signatures in CTCs. Our previous data also indicated that only one-third (6/20, 30%) of patients with non-metastatic high-risk prostate cancer may be able to fully benefit from a synergistic ADT/radiotherapy treatment. However, a 2-month post-RT time point cutoff was too early to conclude disease outcome in our previous study and for a complete assessment of the effects of ADT and RT on 3D telomere architecture of CTCs, those patients need to be followed up for longer period.

Therefore, in the current study, we assessed if the 3D CTC telomere profiles can predict response to treatment when compared with PSA (the standard evaluation). We are comparing different PSA end points as early surrogates for tumor response, such as six-months PSA end levels after ADT, six-month PSA end levels after RT, and twelve-months PSA end levels after completed ADT (+36 months). The cutoff values were chosen on the basis of previous reports in which PSA end levels above or equal to 0.1 ng/mL after radiotherapy and long-course androgen deprivation therapy are associated with an increased risk of recurrence [6,25,26,27].

## 2. Results

### 2.1. High-Risk Prostate Cancer CTCs were Selected Based on Their Androgen Receptor Staining

**Figure 1:**
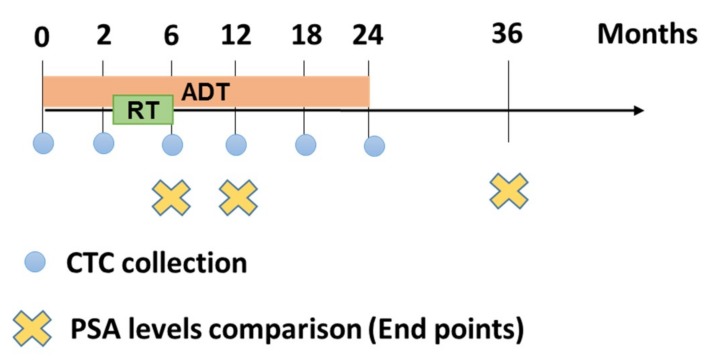
Summary timeline of treatment and PSA/CTC collection points over the course of the study. CTCs were collected and analyzed at 0 m (untreated), 2 m, 6 m, 12 m, 18 m and 24 months and PSA end levels at 6 months ADT, 6 months after finished RT and 36 months after initial treatment were used as early surrogates of treatment response.

One hundred PCa patients had their CTCs collected every six months for 2 years (Figure 1). The CTCs were collected using a size-based filtration technique (ScreenCell), which allows for the isolation of PCa CTCs in patients with low-, intermediate-, and high-risk disease [28]. Figure 1 shows a timeline summary of the treatment and PSA/CTC collection points over the course of the study. CTCs were collected and analyzed at different time points until 24 months and PSA levels at 6 months after continued ADT, 6 months after finished RT, and 12 months post-initial treatment (36 months) were used as early surrogates of treatment response.

**Figure 2:**
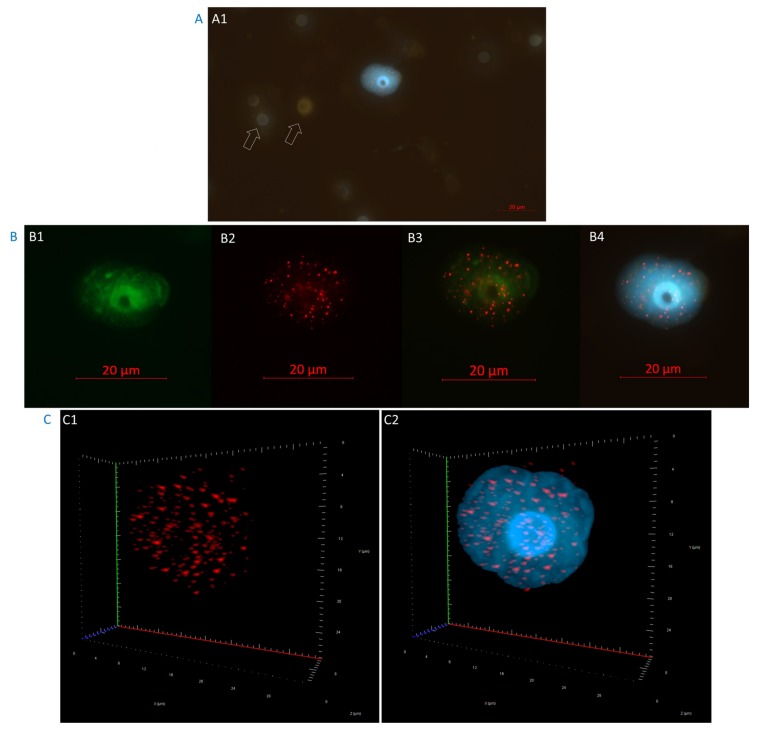
Example of a circulating tumor cell from a high-risk localized prostate cancer patient captured on top of a filter pore (**A**) (The arrows show empty filter pores). The prostate cancer CTCs are recognized based of their AR positive staining (**B**). (**B1**) Two-dimensional image showing a CTC AR+ in FITC (green); (**B2**) CTC with the telomeres labeled with telomere-specific Cy3-labeled probe (red); (**B3**) Merge between FITC and telomeres; and (**B4**) CTC counterstained with DAPI in blue. In C (**C1** and **C2**), the same cell is shown in three-dimensional representation. Red spots represent telomere signals; and the blue is DAPI.

We identified the prostate cancer CTCs based on their positive immunostaining for androgen receptor (AR). All analyzed samples contained androgen receptor-positive cells (Figure 2). Figure 2A shows an isolated CTC stained with AR antibody conjugated with Alexa Fluor 488, AR stains both the intracytoplasmic region and the cell membrane. The expression of AR on the isolated CTCs was found to be heterogeneous. We observed both intersample and intrasample variability in the level of fluorescent intensity. Our results were consistent with those described previously [19]. The AR expression can change during ADT treatment and a mixed population of CTCs with different expression levels of AR can also be found [29]. 

### 2.2. CTCs Dynamics of the 3D Telomere Architecture Stratified Patients into 5 Distinct Subgroups

**Figure 3:**
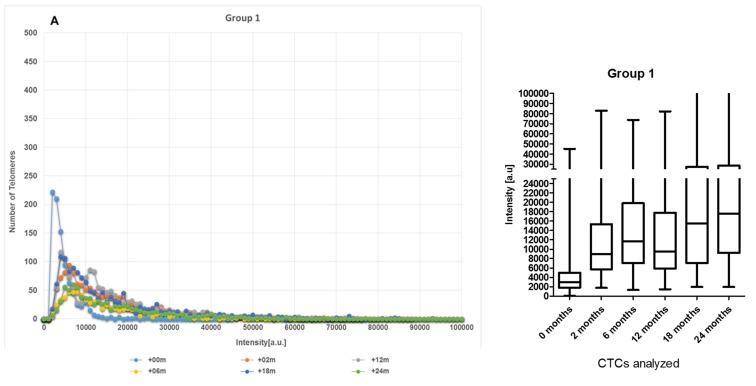

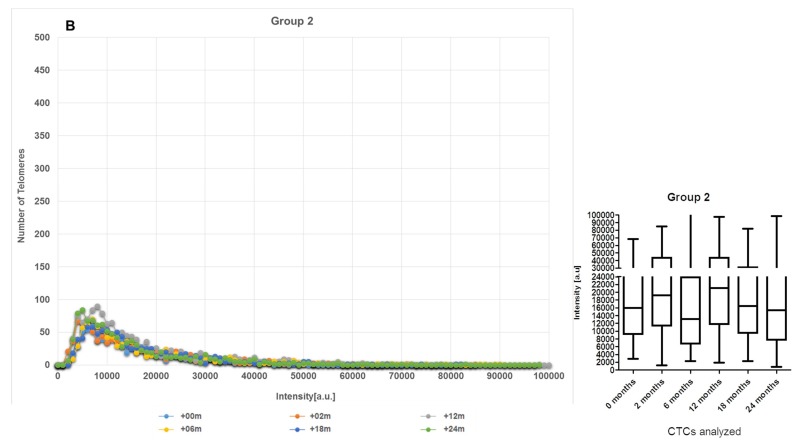

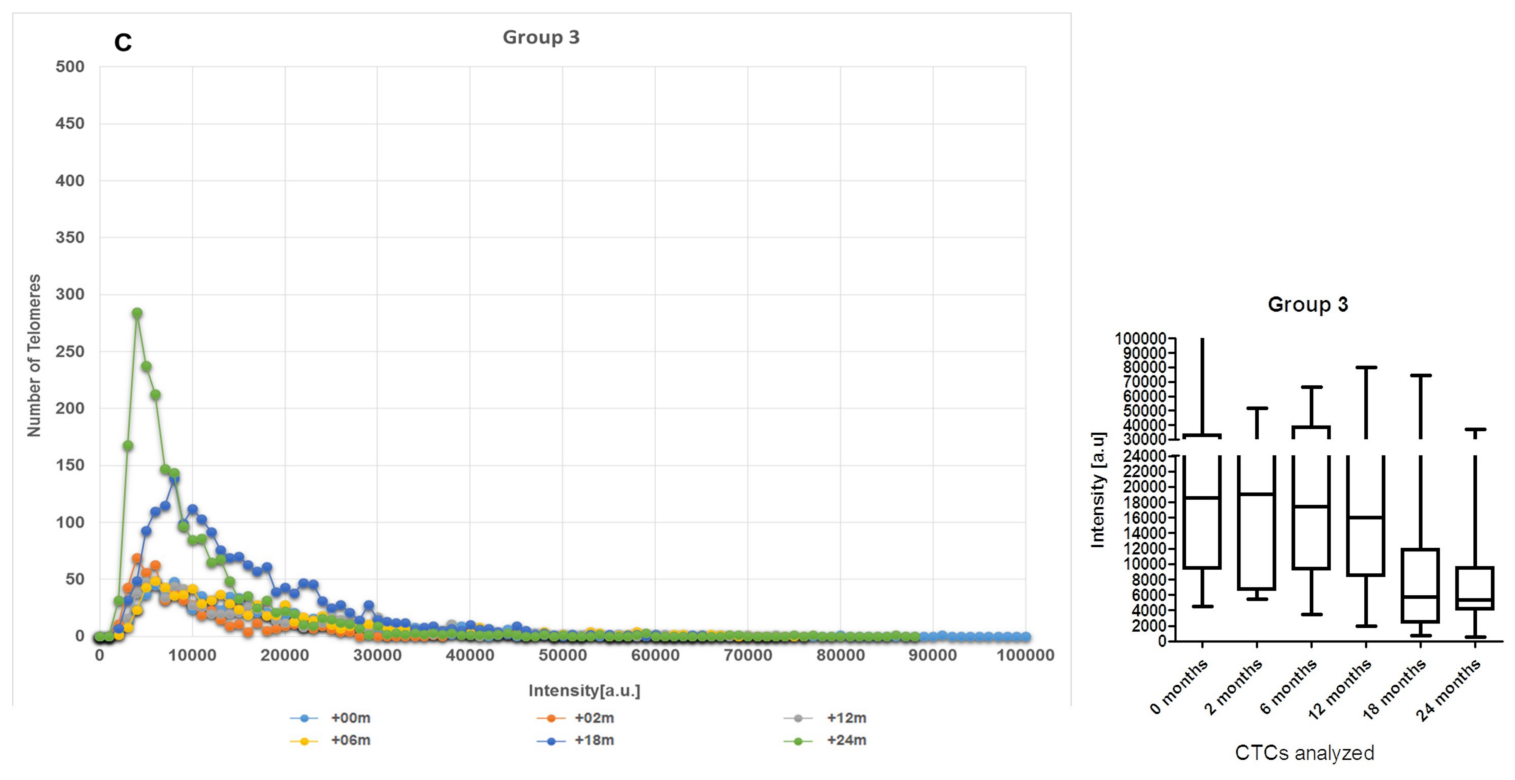

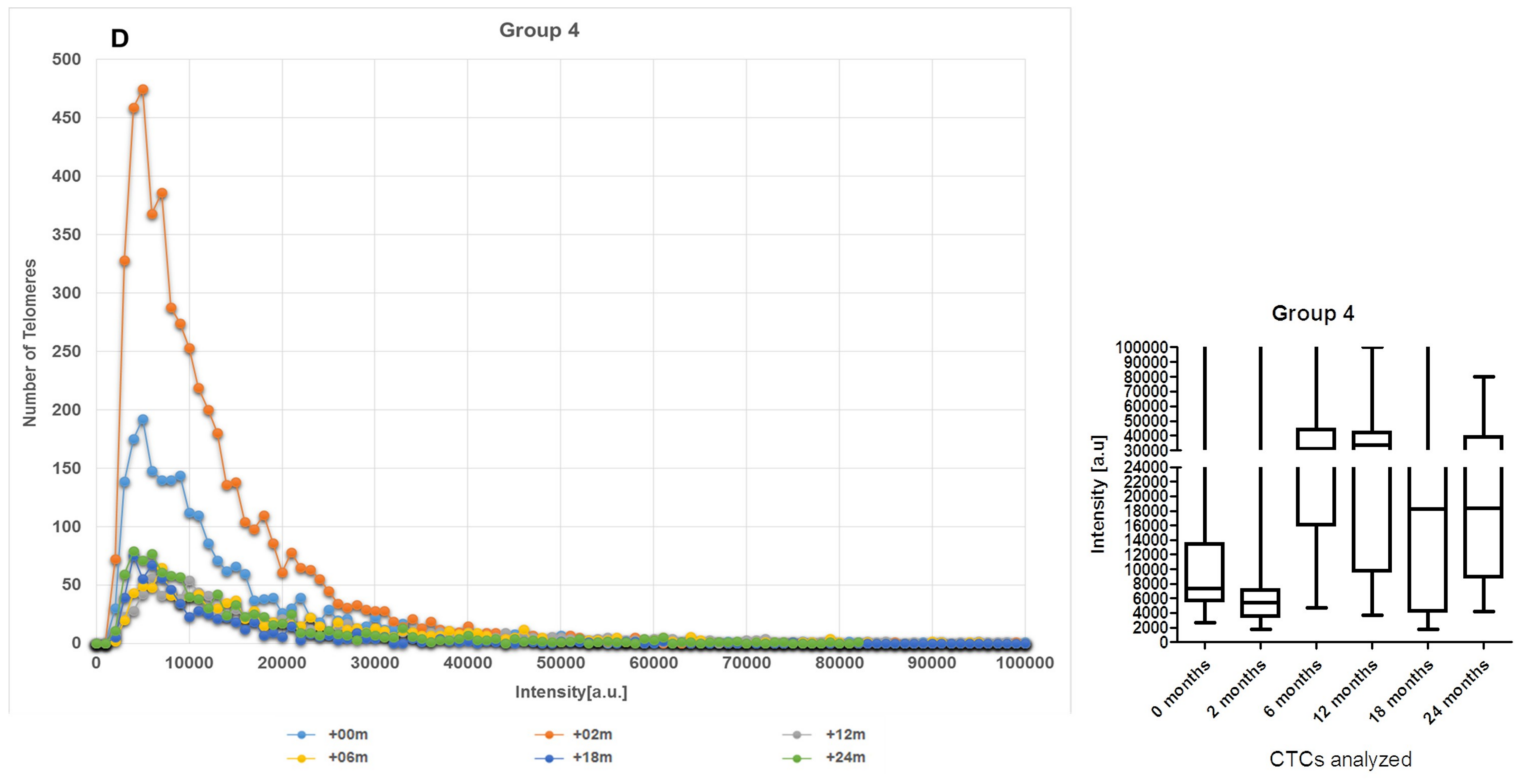

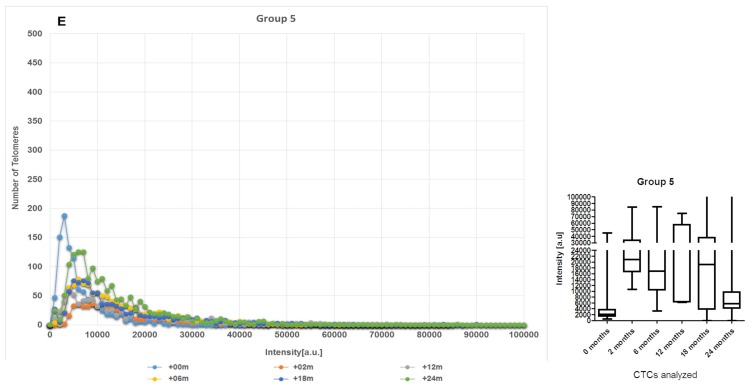
(**A**) Representative examples of the CTCs dynamics of telomere length profiles over time for patients assigned to Group 1 (**A**), Group 2 (**B**), Group 3 (**C**), Group 4 (**D**), and Group 5 (**E**). In each graph, the telomere length is shown in arbitrary units of fluorescence (AU). Baseline profile (+0 month, untreated) and other time point (2, 6, 12, 18, 24 months) are demarked with colors. Bars plot were used to illustrate inter-sample variability of representative individual samples in the groups.

Cancer cells commonly exhibit genomic instability with the telomeres often being shorter than those in normal cells [27,30,31]. Based on the dynamics of the 3D telomere architecture of their CTCs (changes in number of short telomeres over time) at +0, +2, +6, +12, +18 and +24 months, patients were classified into five groups. In Figure 3, telomere length (signal intensity, x-axis) is plotted against the number of telomeres (y-axis) for all CTCs analyzed at each time point. Signals are grouped by their intensity level and this gives a picture of the CTCs telomere distribution in each sample or time point. In Figure 3A, at +0 month, approximately 220 telomeres have the same intensity, less than 10,000 a.u (arbitrary units of relative fluorescence intensity), which is of relatively low intensity and can be correlated with short telomeres. For normal lymphocytes, for example, this plot usually has small peaks between 0 and 20,000 a. u, in which the number of telomeres per nucleus on the y-axis ranges between 5 and 25 [16] and most of the telomere signals have high relative intensities, with signals detected up to 120,000 a.u [16].

Two Teloview^TM^ parameters were used to analyze the CTCs dynamics over time-number of telomere vs telomere length/intensity [32]. The first patient group (Group 1), at baseline (+0 month, untreated), showed a large number (peak count 220) of shorter telomeres (< 10,000 AU) which started to decrease post-ADT (+2 months) and remained at this level from post-RT (+6 months) until 24 months (Figure 3A). The second patient group (Group 2) showed CTC with a moderate number (peak count between 80 and 90) of shorter telomeres at baseline (+0 month) and the telomere length remained stable before and after treatment (Figure 3B). Patient from group 3 displayed CTC with small number (peak count between 40 and 50) of shorter telomeres at baseline (+0 month) and remained stable during all treatment procedures. However, in this group, the number of short telomeres started to increase at +18 months and reached a high peak at +24 months (Figure 3C). The fourth group (Group 4), at baseline (+0 month, untreated), showed a small to moderate number of short telomeres at baseline (+0 month). Nevertheless, when ADT started at +2 months, the effects were shown with a temporally increase of short telomeres that does not continue until the next time point (+6 months) and the number of short telomeres remained low until 24 months (Figure 3D). Patients from our last group (Group 5) displayed CTC with high number of short telomeres at baseline (+0 month) with a peak count between 180 and 200, which decreased during and after treatment. However, the number of short telomeres in Group 5 started to increase after 24 months (Figure 3E). Only patients in Group 2 showed no changes in 3D telomere architecture in response to ADT plus RT. Groups 1 and 4 presented the best dynamics of CTCs with an increase of telomere length in a long-term follow-up and, consequently, decrease of genomic instability. Even though Group 4 showed a peak of short telomeres at +2 months, this is probably due to the start of the ADT therapy, since ADT can induce telomere breaks and telomere fusion [20]. The number of short telomeres continued at low intensities until 24 months. Patients from Groups 3 and 5 exhibited the worst dynamics of CTCs after 24 months, i.e., both presented a high peak of short telomeres at the last time point (+24 months). Supplementary Table S1 shows all patients classified in the five subgroups. The most prevalent being Group 2 with 30%, followed by Group 1 with 25%, Group 5 with 18%, Group 3 with 15% and Group 4 with 12%. Supplementary Figure S1 shows the inter-sample variability of representative individual samples. Lymphocytes for each patient were used as an internal control (Supplementary Figure S2), and representative 3D images for each time point are shown in the Supplementary Figure S3.

### 2.3. The Telomere Parameters Can Predict PSA Increase at 6 Months of ADT, RT and at 36 Months after Initial Therapy

**Figure 4:**
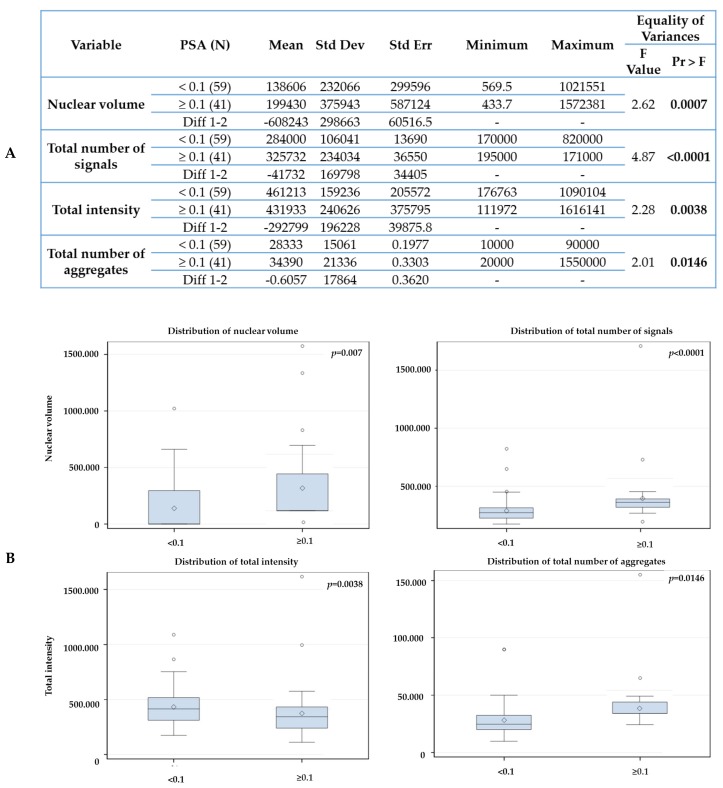
Statistical analysis comparing the telomeres parameters at 0 month with PSA end value at 6 months after androgen deprivation therapy using 0.1 ng/mL cutoff (**A**). (**B**) Box-plots generated by Statistical Analysis Software v 9.4 (SAS, Cary, NC USA). The box is divided in the following way: —the median is the middle line, the 50th percentile- the top of box is the 75th percentile- the bottom box is the 25th percentile. Concerning the whiskers, the upper top of whiskers represent the max observation 1.5× (interquartile range (IQR)—75th percentile minus 25th percentile), while the bottom of whiskers represent the minimum observation 1.5× (1QR from 25th). The observations plotted are outliers—beyond the 1.5× IQR or below. The sign in the box is the mean. The box indicates where 50 percent of the observations lies, extending to the whiskers indicates where most of the data lies and the points outside are extremes.

First, we compared the PSA end values of two time points: after 6 months of ADT and post 6 months of completed RT. Our aim was to identify which baseline telomere parameter (+0 month, untreated) could predict response to treatment. We used 6 months PSA end values (after ADT and after RT) and 36 months after initial therapy as an early surrogate for treatment response. We compare for example, patients with PSA end values below 0.1 ng/ml at 6 months (ADT or RT) vs the group of patients which PSA end value ≥ 0.1 ng/mL. Fifthy-nine patients (59/100) had a stable or decrease in PSA end values (< 0.1 ng/mL) and forty-one had an increased PSA end value above 0.1 ng/mL (≥ 0.1 ng/mL) at 6 months after ADT (Figure 4). Figure 4 shows a comparison between the two groups. The group with PSA ≥ 0.1 ng/mL had significantly higher nuclear volume (*p* = 0.0007), increased total number of signal (*p* < 0.001)—which can indicate an increase of aneuploidy- and increased number of telomere aggregates (*p* = 0.01)—which represent more telomere fusion- in comparison to the group with PSA < 0.1 ng/mL at 6 months after ADT. The total fluorescent telomere intensity, which is equivalent to telomere length, also decreased (*p* = 0.0047) in the group with PSA ≥ 0.1 ng/mL.

Using the 6 months PSA end value after RT as an early surrogate for treatment response, 78% of the patients (78/100) had PSA end value below the 0.1 ng/mL threshold at 6 months after RT (< 0.1 ng/mL) and 22% of the patients (22/100) had PSA end values above the 0.1 threshold (≥ 0.1 ng/mL).

**Figure 5:**
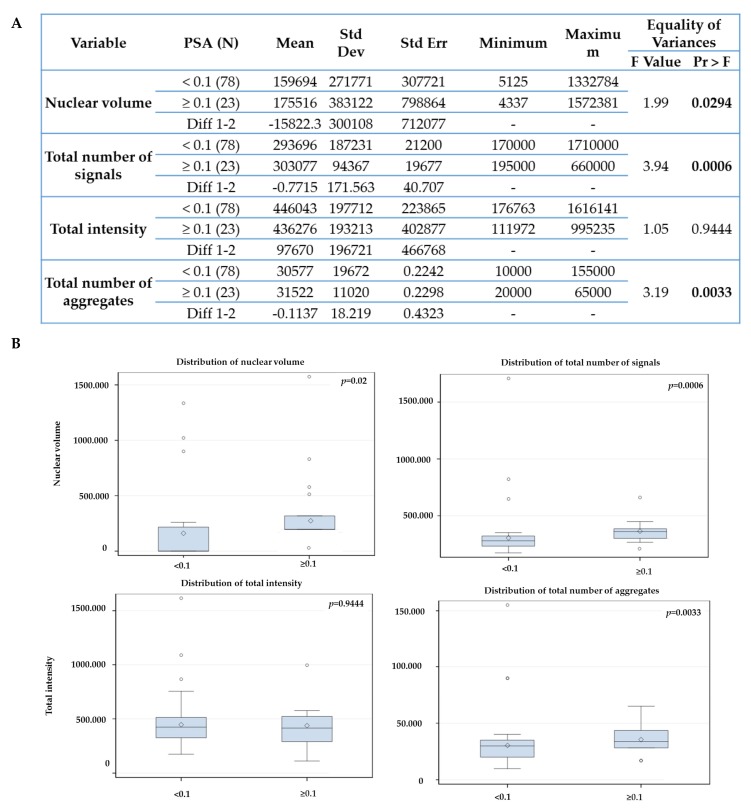
Statistical analysis comparing the telomeres parameters at 0m with PSA end value at 6 months after radiotherapy using 0.1 ng/mL cutoff (**A**). (**B**) Box-plots generated by Statistical Analysis Software v. 9.4. The box is divided in the following way: —the median is the middle line, the 50th percentile- the top of box is the 75th percentile- the bottom box is the 25th percentile. Concerning the whiskers, the upper top of whiskers represent the max observation 1.5× (interquartile range (IQR)—75th percentile minus 25th percentile), while the bottom of whiskers represent the minimum observation 1.5×(1QR from 25th). The observations plotted are outliers—beyond the 1.5× IQR or below. The sign in the box is the mean. The box indicates where 50 percent of the observations lies, extending to the whiskers indicates where most of the data lies and the points outside are extremes.

In Figure 5, we compare the telomeres parameters at 0 month (untreated) with the PSA groups. The nuclear volume (*p* = 0.02), total number of telomere signals (*p* = 0.0006) and formation of telomeres aggregates (*p* = 0.003) also increased in the group with PSA end ≥ 0.1 ng/mL after RT. However, the total intensity did not decrease significantly. In addition, we found no association between the PSA end values and the 3D telomere groups (CTCs dynamics) over time (*p* = 0.38). 

**Figure 6:**
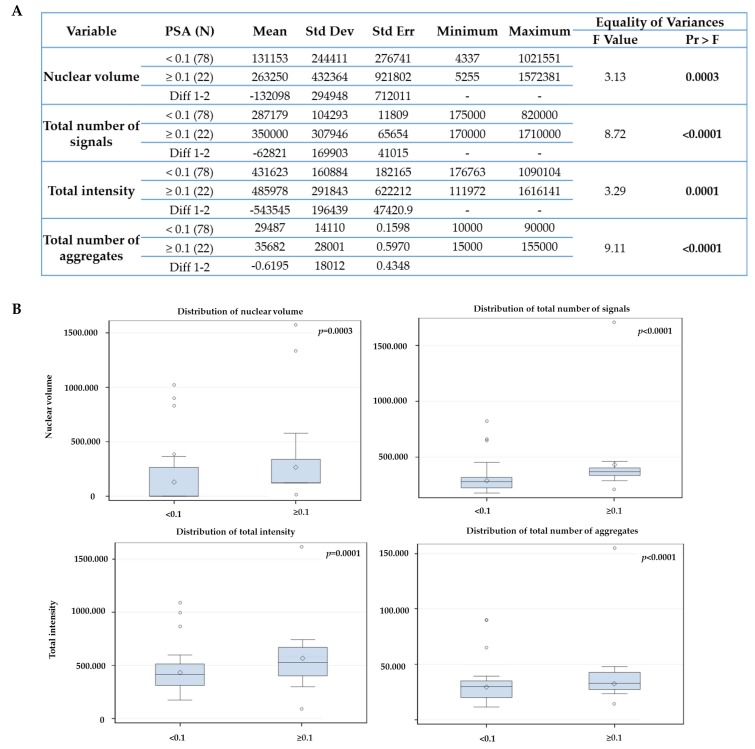
Statistical analysis comparing the telomeres parameters at 0 month with PSA value at 36 months after initial therapy using 0.1 ng/mL cutoff (**A**). (**B**) Box-plots generated by Statistical Analysis Software v. 9.4. The box is divided in the following way: —the median is the middle line, the 50th percentile- the top of box is the 75th percentile- the bottom box is the 25th percentile. Concerning the whiskers, the upper top of whiskers represent the max observation 1.5× (interquartile range (IQR)—75th percentile minus 25th percentile), while the bottom of whiskers represent the minimum observation 1.5× (1QR from 25th). The observations plotted are outliers- beyond the 1.5× IQR or below. The sign in the box is the mean. The box indicates where 50 percent of the observations lies, extending to the whiskers indicates where most of the data lies and the points outside are extremes.

The 36-month PSA end after initial treatment, which means one year after the treatment was completed, was used as an early surrogate for tumor response. 78% of the patients (78/100) had PSA end value below the 0.1 ng/mL threshold at 36 months (< 0.1 ng/mL) and 22% of the patients (22/100) had PSA end values above the 0.1 threshold (≥ 0.1 ng/mL). In Figure 6, we compare the telomeres parameters at 0 m (untreated) with the PSA groups. The nuclear volume *(p* = 0.0003), total number of telomere signals (*p* < 0.0001), and formation of telomeres aggregates (*p* = 0.0001) increased in the group with PSA ≥ 0.1 ng/mL after 36 months of initial therapy. However, the total intensity significantly increased this time (*p* = 0.0001). We attribute the changes in total intensity to the increase in the number of telomere signals and formation of telomeres aggregates visualized since the first time point (6 months post-ADT) in the group with PSA end value above to ≥ 0.1 ng/mL. 

**Figure 7:**
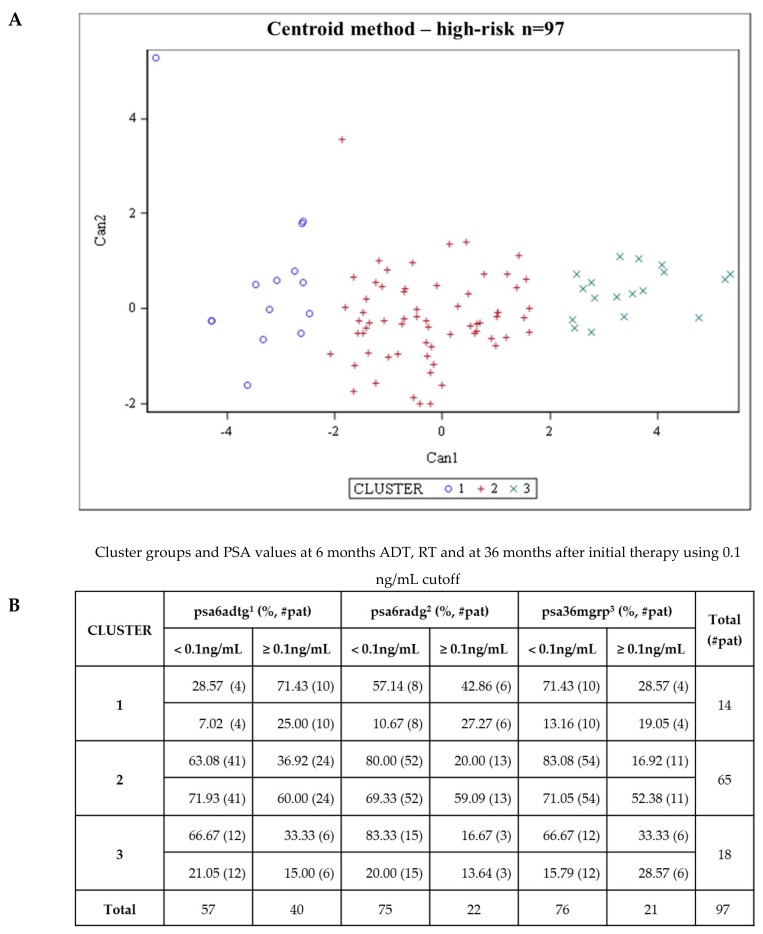
Centroid cluster analysis of 3D nuclear profiling of CTCs from 97 patients with high-risk prostate cancer (**A**). The combination of telomere parameters (Materials and Methods) allows the stratification of patients into clusters. Each cluster possesses a different level of genomic instability and different risk of future prostate mortality, based on their PSA end values after 6 months of ADT 6 months of RT, and 36 months after initial treatment (**B**). Patients in cluster 3 (green) had the highest percentage of patients with PSA ≥ 0.1ng/mL after treatment, while those in cluster 2 (red) and cluster 1 (blue) had an intermediate- to low percentage of patients with PSA ≥ 0.1ng/mL after treatment.

Second, we performed a hierarchical centroid cluster analysis, which combines all TeloView^TM^ data for each patient. The TeloView^TM^ data includes all parameters (nuclear volume, total number of signals, total intensity and total number of aggregates) provided for each telomere. Three patients were excluded from the analysis. The remaining ninety-seven patients were grouped into three subgroups (clusters 1-3) (Figure 7). The three clusters identified from 3D telomere profiling data, after hierarchical centroid cluster analysis, distinguished patients with different levels of genomic instability and different risk of future prostate mortality, based on their PSA end values after 6 months of ADT, 6 months of RT, and 36 months after initial treatment (Figure 7). Supplementary Table S1 shows all patients classified in the three clusters. Cluster 3 contains predominantly high-risk patient for future prostate mortality. Cluster 3 had approximately 30% of patients with PSA end value above 0.1 ng/mL after 6 months of ADT which decreased to 16.67% after RT and return to 30%, after 36 months of initial treatment. In contrast, clusters 1 and 2 had lost the fluctuation in their PSA end values after ADT, RT, and 36 months of ADT. Cluster 3 comprises 18.55% of the patients in this study. Supplementary Table S1 shows all patients classified into the three clusters. Additionally, patients classified as clusters 1, 2, and 3, had different disease aggressiveness (cluster 3 > cluster 1 > cluster 2) based on their genomic instability pattern detected by 3D telomere analysis. 

## 3. Discussion

In most cancers, early detection allows for improved outcomes, but for localized high-risk prostate cancer patients, early detection can also result in overdiagnosis and overtreatment [29]. In addition, localized high-risk prostate cancer patients face a more serious problem, a reliable prognostic tool capable of predicting whether the cancer will eventually develop into a lethal metastatic disease [18]. Although PSA levels are used for disease monitoring, the predictive value of PSA testing and screening is low (around 35%) and have been associated with a high rate of overdiagnosis/overtreatment in clinical trials [32,33,34]. In addition, other clinical parameters such as clinical stage and Gleason score tumor grade have limitations to detect and predict disease outcome [35]. This scenario demonstrates the importance for novel and less-invasive biomarkers that can reliably identify patients at high risk for recurrence and metastases.

Telomere shortening is one of the earliest events in prostate cancer tumorigenesis and continue during tumor progression [18]. Since detection of shorter telomeres is associated with increased occurrence of lethal prostate cancer and decreased survival times, the 3D telomere assessment could potentially improve prostate cancer screening by adding, to the current approaches, prognostic information to better stratify patients requiring active surveillance or more definitive treatment, such as surgical castration [18,31]. 

To our knowledge, the current study is the first to investigate the dynamics of the nuclear 3D telomere architecture in CTCs derived from patients with non-metastatic high-risk prostate cancer before, post-ADT, and post-RT (until 36 months after initial treatment). CTCs isolated before treatment was divided into five distinct telomere signatures. Remarkably, CTC analysis showed distinct dynamic changes in their 3D telomere signatures, which were unique to each group during ADT+RT treatment. Recent studies have provided insights to the clinical value of CTCs collected from blood in prostate cancer [17,36,37,38]. In the present study, we have used the ScreenCell filter device, which allows a size-based separation of CTCs from whole blood of patients with non-metastatic high-risk prostate cancer [28]. Captured CTCs underwent 3D telomere analysis to determine their nuclear 3D telomere profile before treatment and to investigate dynamic changes to their 3D telomere profiles during and after treatment with ADT and RT. The CTCs can be detected in blood before the occurrence of clinically relevant metastases providing insight into the genetics of the primary prostate tumor [39,40,41,42,43,44,45]. In this study, we show that nuclear 3D telomere architectural analysis is highly sensitive in detecting telomere changes that affect the genome stability in CTCs and may prove to be a valuable tool in monitoring treatment response in patients with non-metastatic high-risk prostate cancer. 

As stated above, in a previous study, we showed the usefulness of PSA serum levels and CTC blood counts after treatment [17]. In studies using the CellSearch® system to enrich and isolate CTC in localized high-risk prostate cancer, no correlation between CTC count and other clinical-pathological parameters was found [46,47,48]. This highlights the importance of molecular analysis of CTC instead of only CTC count to provide additional information about the tumor. For example, AR-V7 expression in CTCs was found to be a predictor of response for treatment with abiraterone/enzalutamide and disease outcome [46].

Here, we assessed the effects of ADT and RT on 3D telomere architecture of captured CTCs until 24 months. We found that the 100 high-risk patients could be stratified into five distinct telomere signatures based on telomere numbers and telomere length (intensity). Furthermore, each of the five CTC groups responded to the combined treatment with different changes to telomere profiles, thus, providing unique insight about the complexity by which high-risk prostate cancer cells can adapt to those treatments. We also showed in previous studies the potential of 3D telomere architecture in predicting disease outcome and patient survival [16,17,47,48,49]. Based on our 3D telomere analysis, Group 2 may qualify as a non-responder, since the telomere profiles were stable throughout treatment. However, for Group 1, the treatment resulted in a dramatic decrease in the number of telomeres with an intensity less than 10,000 a.u. Intriguingly, this 3D telomere profile remained unaltered despite radiation-induced cell death. For Group 4, all patients presented a peak after ADT started, which decreased in later time points. Zhou et al. have already demonstrated that AR inactivation by androgen deprivation, in LNCaP cells (prostate cancer cell line) can induce telomere breaks and telomere fusion [20]. The peak that we observed in +2m can be a consequence of the ADT treatment, which induce genomic instability and consequently death. Similar to Groups 1 and 4, the treatment for Groups 3 and 5 also had the number of short telomeres decreased. However, at later time points, the population with short telomeres started to increase reflecting a positive selective pressure of ADT plus RT in favor of resistant prostate cancer clones. 

The ability of cancer cells to survive specific treatments, such as ADT and RT, involves changes in 3D telomere architecture and reflects the effects of complex cellular processes in which genomic stability, instead of causing death, ensure tumor cell survival. The effect of ADT plus RT on specific 3D telomere profiles may reflect the evolution of heterogeneous prostate tumor sub clones, as showed at later time points for Groups 3 and 5. The cellular mechanisms responsible for the dynamic telomere alterations in these patients are currently unknown. It is important to recognize that the observed heterogeneity in telomere phenotype was limited to five unique 3D telomere signatures in 100 localized high-risk patient samples. The effect of these profiles on patient survival awaits future analysis. However, we used PSA after 6 months of ADT, after 6 months of finished RT, and 36-months after initial treatment as an early surrogate for tumor response. In all time points, increase of nuclear volume, total number of signals, and total number of aggregates were significantly different between the two patient population (< 0.1 ng/mL and ≥ 0.1 ng/mL PSA end). The cutoff value was chosen on the basis of previous reports in which increasing levels of PSA above 0.1 ng/mL after ADT and radiotherapy was associated with an increased risk of recurrence [6,22,23,24,25]. Additionally, the total intensity (associated with telomere length) decreased at 6 months of continued ADT and increased after 36 months after initial treatment. We attributed this to a process where the decrease of telomere length leads to a decrease of total intensity; however, as the formation of telomere aggregates (clusters of telomeres) continues, the resulting high intensity values of these clusters ensued an increase in the total intensity measurements in 36 months. It is important to highlight that we compared our data with the only approved biomarker guiding for treatment decisions in PCa [7]. However, PSA values often do not represent the current tumor status, potentially misleading therapeutic decisions [7].

Nevertheless, we found no association between the PSA end values and the 3D profile with CTCs dynamics over time (*p* = 0.38). Our data also predicted that only 32% (Groups 1 and 4) of the patients with non-metastatic high-risk prostate cancer could benefit from the combination of ADT/radiotherapy treatment. Additionally, 3D telomere analysis of circulating tumor cells offers a non-invasive method to follow up prostate patients during their treatment cycle. 

The centroid cluster analysis identified three clusters, using all Teloview^TM^ parameters (number of telomeres, total intensity/length, telomere aggregates, and nuclear volume), which separated patients with different levels of genomic instability. Cluster 3 seems to correspond to somewhat more aggressive phenotypes than Cluster 2 and 1. We observed that in PSA 6 months after ADT, 33.33% of patients had PSA values above 0.1 ng/ml. This percentage decrease in the second time point (PSA 6 after RT), however, in the third time point (PSA after 36 months), cluster 3 is the only group that return to the same scenario found in the first time point (PSA 6 months after ADT), with 33.33% of the patients above 0.1 ng/ml (PSA). In the other clusters (1 and 2), the percentage of patients above 0.1 ng/ml after 36 months of treatment is lower than previous time points. In spite of a 3-years follow-up be too short to detect prostate cancer related mortality, our results demonstrate that CTCs retain important genetic information that could be used as a real time liquid biopsy to guide therapeutic decision and avoid overtreatment. 

The current study has two important limitation. First, the 3-years follow-up was too short to detect prostate cancer related mortality, which affects the correlation of our data with a “real” clinical end-point. Second, our results were correlated with PSA end levels. Although PSA measurement after radiotherapy and androgen deprivation for localized prostate cancer has been proposed as an early prognostic biomarker [50,51]. PSA positive predictive value is only 25–40% [52]. We found that our telomere parameters are significantly different between PSA groups, which highlights the potential of our biomarker to be equal or superior to PSA. However, the effect of our findings on patient survival still awaits future analysis.

## 4. Materials and Methods 

### 4.1. Patient Samples, Treatment and Study Design

The study cohort included one hundred (100) men who were treated between 2014 and 2016 with long-course ADT combined RT for clinically localized and non-metastatic high-risk prostate cancer. High-risk prostate cancer patients defined as having either cT3, Gleason score 8–10, or PSA > 20 ng/mL with no history of prior ADT, RT, or chemotherapy. Tumors were considered non-metastatic in patients with negative results in bone scan (Tc-99m-methylene diphosphonate (MDP)) and CT of the abdomen, and pelvis). This study was approved by the University ethics committee (University of Manitoba Ethics Protocol Reference No. H2011:336). All men were initially treated with 24 months of androgen deprivation therapy in the form of oral bicalutamide (given at a dose of 50 mg every day) (Figure 1) in combination with injections of goserelin or leuprolide. 

Radiotherapy doses were 78 Gy after 2 months of initial therapy with ADT (for 3 months). This study was design for a clinical assessment and laboratory testing (CTCs collection plus PSA) in different time points: 0, 2, 6, 12, 18 and 24 months, being 0 m at diagnosis (Figure 1). All patients outside this time interval were excluded. We consider as an end-point PSA level < 0.1 ng/mL vs. ≥ 0.1 ng/mL after 6 months of ADT, after 6 months of RT and after 12 months of complete ADT (+36 months).

### 4.2. CTC Isolation and Androgen Receptor (AR) Staining

Patient blood was processed using the ScreenCellR filter method for the separation of prostate CTC [28]. Briefly, 3 mL of patient blood were incubated with 4 mL of ScreenCell buffer for 8 min. This buffer lyses the red blood cells, prefixes all nucleated cells present in the blood sample while preserving their architecture and enabling their fixation onto the filter membrane of the device. Thereafter, this mix is filtrated passing through the microporous membrane filter (7.50 m pore size). This technique results in an average of 91.2% recovery CTC rate of 91.2% [28].

Filters containing captured cells were fixed for 10 min in 3.7% formaldehyde/1× PBS (Sigma, Oakville, ON, Canada) at room temperature. Filters were washed 3× 5 min each in 1× phosphate buffered saline (PBS)/50 mM MgCl_2_ and blocked at 37 °C for 30 min in 4× SSC (0.6 M NaCl; 0.06 M sodium citrate)/4% bovine serum albumin (BSA, all Sigma-Aldrich, St. Louis, MO, USA). A FITC labelled mouse monoclonal antibody raised against amino acid residues 299-315 of the human androgen receptor (AR-441; Santa Cruz Biotechnology, Dallas, Texas, USA) was applied at 1:50 (20 ng/L) and allowed to incubate for 10 min at 37 °C in a humidified chamber. Excess antibody was removed in 3× washes with 1× PBS/50 mM MgCl_2_ for 5 min each at RT. Filters were dehydrated in an ethanol series (70%, 90%, 100%), air dried and attached to microscopy slides with clear nail polish. Slides were then counterstained with DAPI (Sigma-Aldrich) and mounted with Vectashield (Vector Laboratories, Burlington, ON, Canada).

### 4.3. Telomere Three-dimensional Quantitative Fluorescent in situ Hybridization (3D-QFISH)

For 3D-QFISH [53], cells on the filters were incubated in 1× PBS for 5 min followed by a 10 min fixation in 3.7% formaldehyde/1× PBS and 3× washes in 1× PBS for 5 min each. Filters were treated with (50 g/mL pepsin (Sigma) in 0.01M HCl for 10 min at 37 °C, 1× washed in 1× PBS for 5 min followed by post-fixation for 10 min in 3.7% formaldehyde/1× PBS and 3× washes in 1× PBS for 5 min each. Filters were dehydrated in an ethanol series and air dried. Fluorochrome-coupled (Cy3) Telomere specific peptide nucleic acid (PNA) probe (DAKO, Agilent Technologies, Santa Clara, CA, USA) was applied (5 μL probe/slide) and, following denaturation at 80 °C for 3 min, hybridization was done for 2 h at 30 °C. Slides were washed in 70% deionized formamide (Sigma-Aldrich) in 10 mM Tris pH 7.4 for 15 min, rinsed in 1× PBS and once each in 2× SSC (5 min at 55 °C), 0.1× SSC and 2× SSC/0.05% Tween-20 at RT. Filters were again dehydrated and air dried. Filters were removed from the metal support ring using an 8 mm biopsy punch, placed on a new slide, DAPI stained, mounted with Vectashield (Vector Laboratories) with a coverslip.

### 4.4. Imaging & Analysis

Slides were imaged on a Zeiss AxioImager Z2 microscope with a Zeiss AxioCam MRmm Rev 3 digital camera using AxioVision Release 4.8.2 (Zeiss, Jena, Germany). A Cy3 filter was used to detect the Cy3 probe nuclear hybridization to telomeric repeats at an exposure time of 500 ms for all samples examined. A FITC filter was used to determine the presence of AR antibodies. Exposure times for the DAPI filter differed between slides. Eighty focal planes spaced 200 nm apart were imaged to create a three dimensional nuclear images of the circulating tumor cells and lymphocytes on the filter. Images were deconvolved using a constrained iterative algorithm [32]. For each patient sample, 30 CTC and 30 lymphocyte nuclei were analyzed using TeloView^TM^ software [33] (used with permission of Telo Genomics Corp Inc. Toronto, ON, Canada). Each cell was analyzed for intensity of signal, presence of telomere aggregates (two or more signals that cannot be resolved due to proximity and defined as a signal with intensity above the standard deviation of signal intensity for that cell), number of signals per nucleus and nuclear volume. These measurements were determined for CTCs from each patient isolated at different time points during their treatment.

### 4.5. Statistical Analysis

For statistical analysis comparing PSA levels, we set a threshold of PSA end of 0.1 ng/L of PSA at 6 months after continues ADT, 6 months of after completed RT and 36 months after initial treatment. The CTCs were analyzed in five time points (+0, +2, +6, +12 and +24 m). For each time point, thirty cells were ranked and quartiles calculated for each telomere parameter measured by TeloView^TM^ and compared between patients falling on both sides of the threshold. The telomeric parameters (number, length, telomere aggregates, nuclear volume, a/c ratio, etc.) were compared using a nested factorial analysis of variance followed by a least-square means multiple comparison. Graphical presentations indicated the p-value for the overall test of differences across all time points. Chi-square analysis compared the percentage of interphase telomeric signals intensities at defined quartile cut-offs. A significance level was set at 0.05. The Box-plots generated by Statistical Analysis Software v. 9.4 (SAS, Cary, NC USA. 

## 5. Conclusions

Our study identified CTCs with three unique 3D telomere profiles among patients with localized high-risk prostate cancer. The distinct CTC telomere dynamics in each patient group in response to treatment provides a strong rationale for the use of 3D telomere analysis on CTCs as a way to monitor treatment response.

## Supplementary Materials

The following are available online at https://www.mdpi.com/2072-6694/11/8/1165/s1. Table S1: List of all patients with corresponding Gleason score and TMN staging at baseline, PSA levels at diagnosis, corresponding assigned telomere profile grouping and cluster for each patient. Supplementary Figure S1. Representative bar plots to illustrate inter-sample variability of representative individual samples in the Groups 1, Group 2, Group 3, Group 4, and Group 5. Supplementary Figure S2. Representative examples of the lymphocytes (internal control) dynamics of telomere length profiles over time for patients assigned to Group 1, Group 2, Group 3, Group 4 and Group 5. Supplementary Figure S3. Example of a circulating tumor cell from a high-risk localized prostate cancer patient captured on top of a filter pore for each time point. Three-dimensional representation of a CTC with the telomeres labeled with telomere-specific Cy3-labeled probe (red) and Merge between telomeres; and the counterstained DAPI (blue).
