# Supplementary material for: Long-Term Dynamics of Three Dimensional Telomere Profiles in Circulating Tumor Cells in High-Risk Prostate Cancer Patients Undergoing Androgen-Deprivation and Radiation Therapy
Source: Cancers (Basel). 2019 Aug 14;11(8):1165. doi: 10.3390/cancers11081165 (PMC6721586; doi:10.3390/cancers11081165)
Supplement: Supplementary file 1 [file cancers-11-01165-s001.pdf]

Supplementary Table 1: List of all patients with corresponding Gleason score and TMN staging at baseline, PSA levels at diagnosis, corresponding assigned telomere profile grouping and cluster for each patient

Patient	Gleason score (+0m)	TNM staging (+0m)	PSA pre-TRX	PSA 6 months after continued ADT	PSA 6 months after complete radiotherapy	PSA 36 months after initial treatment	Telomere group	Cluster	Can1	Can2
**1**	9	T2bNXMX	11,24	<0.1	<0.1	<0.1	Group 1	2	-147919	-0.52495
**2**	9	T2b	29,18	≥0.1	≥0.1	≥0.1	Group 1			
**3**	9	T2a	13,27	<0.1	<0.1	<0.1	Group 1			
**4**	8	T2b	5,83	<0.1	<0.1	<0.1	Group 2	2	-0.74308	-0.32467
**5**	8	T2a	7,44	<0.1	<0.1	<0.1	Group 5	2	161239	-0.50863
**6**	9	T2a	0,78	<0.1	<0.1	<0.1	Group 2	2	-0.28599	-100708
**7**	9	T2a	41,14	<0.1	<0.1	≥0.1	Group 1	1	-262602	-0.53003
**8**	9	T2c	26,68	<0.1	<0.1	<0.1	Group 4	2	0.51338	-0.37376
**9**	9	T2cN0M0	14,42	≥0.1	≥0.1	<0.1	Group 2	2	-141838	0.19197
**10**	9	T1c	26,14	≥0.1	<0.1	<0.1	Group 1	1	-246658	-0.10748
**11**	8	T1cNXM0	14,09	≥0.1	≥0.1	<0.1	Group 1	1	-258832	183815
**12**	8	T1c	6,93	<0.1	<0.1	<0.1	Group 1	1	-260056	177872
**13**	8	T2b	23,19	≥0.1	<0.1	≥0.1	Group 2	1	-532545	527887
**14**	10	T2cNXM0	14,56	≥0.1	<0.1	<0.1	Group 5	3	282157	0.21655
**15**	9	T2a	12,34	≥0.1	<0.1	≥0.1	Group 2	2	-157354	-0.51530
**16**	8	T2b	40,02	<0.1	<0.1	<0.1	Group 2	2	0.98543	-0.79690
**17**	9	T2c	23,8	≥0.1	<0.1	≥0.1	Group 2	2	-208020	-0.96423
**18**	9	T2b	4,14	<0.1	<0.1	<0.1	Group 2	2	-0.00424	-161753
**19**	8	T2a	15,82	≥0.1	≥0.1	≥0.1	Group 5	1	-346410	0.49974
**20**	9	T2cN0M0	15,41	≥0.1	<0.1	<0.1	Group 2	2	120507	0.71111

**21**	9	T2a	11,26	<0.1	<0.1	<0.1	Group 3	2	0.44128	138811
**22**	8	T2a	11,25	≥0.1	≥0.1	≥0.1	Group 2	2	102142	-0.08522
**23**	8	T1cN0M0	7,49	≥0.1	<0.1	≥0.1	Group 3	3	276172	-0.51295
**24**	8	T3a	26,11	<0.1	<0.1	<0.1	Group 2	2	-100535	-102836
**25**	8	T2a	7,42	<0.1	<0.1	<0.1	Group 1	2	-101953	0.79975
**26**	9	T2bN0M0	17,67	≥0.1	≥0.1	≥0.1	Group 1	2	-0.09862	0.48048
**27**	8	T3NxM0	21,79	<0.1	<0.1	<0.1	Group 1	3	354188	0.31104
**28**	8	T2b	8,3	<0.1	<0.1	<0.1	Group 5	3	262253	0.42097
**29**	8	T1c	10,84	<0.1	<0.1	<0.1	Group 1	3	242184	-0.23919
**30**	8	T1c	21,94	<0.1	<0.1	≥0.1	Group 5	2	-136686	-0.93894
**31**	8	T2a	10,44	<0.1	<0.1	<0.1	Group 1	2	0.77460	0.71060
**32**	9	T2bNXM0	14,25	<0.1	<0.1	<0.1	Group 4	2	-0.41973	-201287
**33**	9	T1c	20,58	≥0.1	≥0.1	≥0.1	Group 5	2	142330	110480
**34**	9	T2b	21,86	<0.1	<0.1	<0.1	Group 2	2	-0.21173	-199808
**35**	9	T2a	22,13	<0.1	<0.1	<0.1	Group 3	2	103076	-0.09941
**36**	9	T1c	11,19	<0.1	<0.1	<0.1	Group 1	2	-187272	356138
**37**	8	T1c	5,54	<0.1	<0.1	≥0.1	Group 4	3	277013	0.53289
**38**	8	T1cNxM0	15,06	<0.1	<0.1	<0.1	Group 5	2	-123398	-157546
**39**	9	T2a	3,08	≥0.1	<0.1	<0.1	Group 2	2	0.70685	-0.31559
**40**	8	T1c	8,05	<0.1	<0.1	<0.1	Group 5	1	-321109	-0.01944
**41**	9	T1c	14,05	≥0.1	≥0.1	<0.1	Group 2	2	0.64119	-0.48360
**42**	8	T1c	5,78	<0.1	<0.1	<0.1	Group 2	2	-165160	-175129
**43**	9	T2b	8,4	<0.1	<0.1	<0.1	Group 3	2	101151	-0.16676
**44**	9	T2cNXM0	26,6	<0.1	<0.1	<0.1	Group 4	2	-0.29527	-0.72122
**45**	8	T1c	13,07	≥0.1	<0.1	<0.1	Group 2	1	-258187	0.55321
**46**	9	T1cN1M0	41,68	<0.1	<0.1	<0.1	Group 1	3	337705	-0.17550

**47**	8	T2a	5,26	<0.1	<0.1	≥0.1	Group 1	3	330126	109388
**48**	8	T2b	11,76	≥0.1	≥0.1	<0.1	Group 2	2	-162858	-119404
**49**	8	T2bN0M0	10,79	<0.1	<0.1	≥0.1	Group 2	3	324360	0.24532
**50**	8	T2b	17,1	≥0.1	<0.1	<0.1	Group 1	3	411922	0.75707
**51**	8	T1cNXM0	16,49	<0.1	<0.1	<0.1	Group 5	2	-112927	0.46203
**52**	8	T1cNXM0	17,11	≥0.1	<0.1	<0.1	Group 5	2	138686	0.42735
**53**	8	T3a	255,2	≥0.1	≥0.1	≥0.1	Group 5	3	408517	0.92432
**54**	9	T2a	15,27	<0.1	<0.1	<0.1	Group 4	2	-0.68257	0.42220
**55**	9	T2a	10,94	<0.1	<0.1	≥0.1	Group 4	2	-118024	0.99691
**56**	9	T2b	12,64	≥0.1	<0.1	<0.1	Group 5	2	-0.47231	-0.01760
**57**	9	T1cNXM0	12,27	<0.1	<0.1	≥0.1	Group 3	2	0.63361	-0.32300
**58**	8	T1CNXM0	8,09	≥0.1	<0.1	<0.1	Group 3	2	-180304	0.02753
**59**	9	T1CN0M0	11	≥0.1	<0.1	<0.1	Group 2	2	0.13262	135275
**60**	9	T1c	51,81	<0.1	<0.1	<0.1	Group 1	1	-274912	0.77449
**61**	9	T1cN0M0	81,48	≥0.1	≥0.1	≥0.1	Group 5	2	-0.82118	-0.95578
**62**	8	T2N0M0	10,61	<0.1	<0.1	<0.1	Group 2	2	0.15314	-0.53915
**63**	9	T1c	21,03	≥0.1	≥0.1	<0.1	Group 5	1	-363115	-160852
**64**	8	T2cN0M0	11,02	<0.1	<0.1	<0.1	Group 4	2	-0.26551	-0.38642
**65**	8	T1cN0M0	28	<0.1	<0.1	<0.1	Group 1	2	0.49166	0.29631
**66**	9	T2bN0M0	11,89	≥0.1	<0.1	<0.1	Group 1	2	-0.71121	0.33931
**67**	9	T1cNXM0	36,41	<0.1	<0.1	<0.1	Group 2	2	0.60510	-0.52022
**68**	8	T2aN0M0	12,68	<0.1	<0.1	<0.1	Group 3	2	-0.53824	-188695
**69**	8	T3N0M0	56,71	<0.1	<0.1	<0.1	Group 3	2	119635	-0.60886
**70**	9	T4N0M0	15,93	<0.1	<0.1	<0.1	Group 5			
**71**	9	T2a	34,42	≥0.1	<0.1	<0.1	Group 3	2	-0.22517	-136017
**72**	9	T2cN0M0	6,6	<0.1	<0.1	≥0.1	Group 5	2	-141647	-0.41423

**73**	9	T2b	9,17	<0.1	<0.1	<0.1	Group 5	2	-0.17163	-117332
**74**	9	T2cN0M0	116,3	≥0.1	≥0.1	≥0.1	Group 4	1	-428965	-0.25561
**75**	9	T2c	33,78	≥0.1	≥0.1	<0.1	Group 3	1	-428965	-0.25561
**76**	8	T2c	5,32	<0.1	<0.1	<0.1	Group 4	3	246297	-0.42132
**77**	8	T2c	0,07	<0.1	<0.1	<0.1	Group 2	2	-0.71630	-0.22374
**78**	9	T1cN0M0	21,03	<0.1	<0.1	<0.1	Group 3	2	-165589	0.65181
**79**	8	T2aN0M0	13,69	<0.1	<0.1	<0.1	Group 3	2	-0.47854	-0.18046
**80**	9	T2a	15,01	≥0.1	<0.1	<0.1	Group 4	1	-307468	0.58844
**81**	8	T1c/T2a	8,43	<0.1	<0.1	<0.1	Group 3	2	0.27889	0.04974
**82**	9	T2b	26,05	≥0.1	≥0.1	<0.1	Group 2	2	-124395	0.54719
**83**	9	T2a	17,79	≥0.1	≥0.1	<0.1	Group 3	2	-0.54808	0.96576
**84**	9	T2bN0M0	24,02	≥0.1	≥0.1	≥0.1	Group 2	2	0.90935	-0.63758
**85**	8	T2a	10,89	<0.1	<0.1	<0.1	Group 2	2	161227	0.00918
**86**	10	T1cN0M0	10,51	≥0.1	≥0.1	<0.1	Group 5	1	-332960	-0.64543
**87**	8	T2aN0M0	11,57	<0.1	<0.1	<0.1	Group 2	2	-136139	-0.30145
**88**	9	T3aN0M0	14,32	≥0.1	<0.1	<0.1	Group 1	2	-147886	-0.08015
**89**	8	T1c	21,09	≥0.1	<0.1	<0.1	Group 2	2	-109072	-0.26580
**90**	9	T3a	8	<0.1	<0.1	<0.1	Group 1	2	152494	-0.20074
**91**	8	T1c	13,19	≥0.1	≥0.1	<0.1	Group 5	2	-154630	-0.25919
**92**	8	T2b	0,01	<0.1	<0.1	<0.1	Group 2	3	250350	0.72792
**93**	9	T1c	8,54	≥0.1	≥0.1	<0.1	Group 4	2	-0.19839	-0.81608
**94**	9	T2c	17,26	≥0.1	≥0.1	<0.1	Group 2	3	373469	0.37421
**95**	8	T1c	25,79	<0.1	<0.1	<0.1	Group 2	2	-0.28999	-0.26258
**96**	8	T1c	15,74	≥0.1	≥0.1	≥0.1	Group 3	3	535144	0.71030
**97**	9	T1c	75,97	<0.1	<0.1	<0.1	Group 4	3	525865	0.61193
**98**	9	T2bN0M0	13,61	<0.1	<0.1	<0.1	Group 1	3	476392	-0.20637

**99**	9	T2aN0M0	20,56	<0.1	<0.1	<0.1	Group 1	3	364578	105203
**100**	8	T1c	42,58	<0.1	≥0.1	<0.1	Group 1	2	156283	0.60847

Group 1

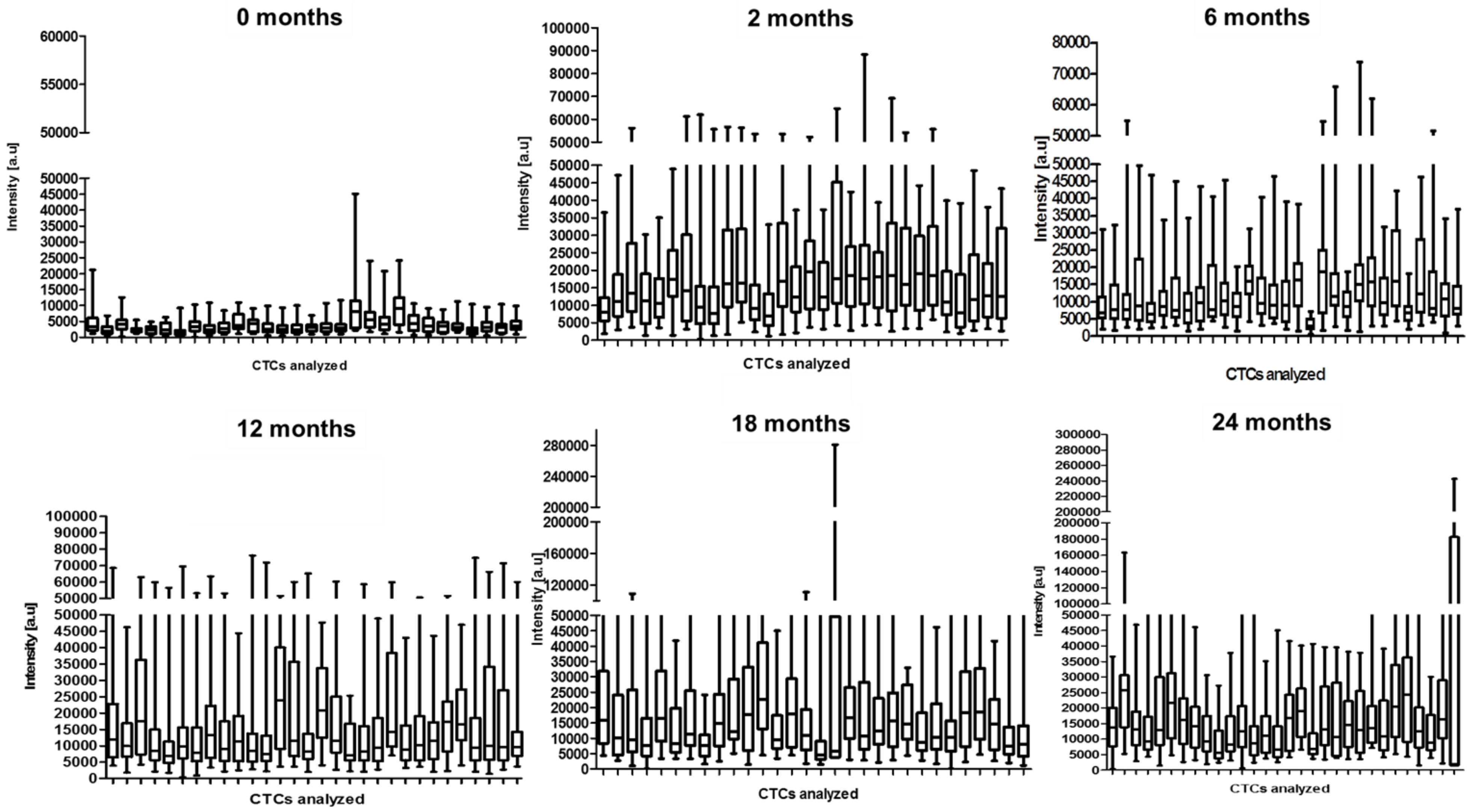
0 months
2 months
6 months
12 months
18 months
24 months
Intensity [a.u]
CTCs analyzed

Group 2

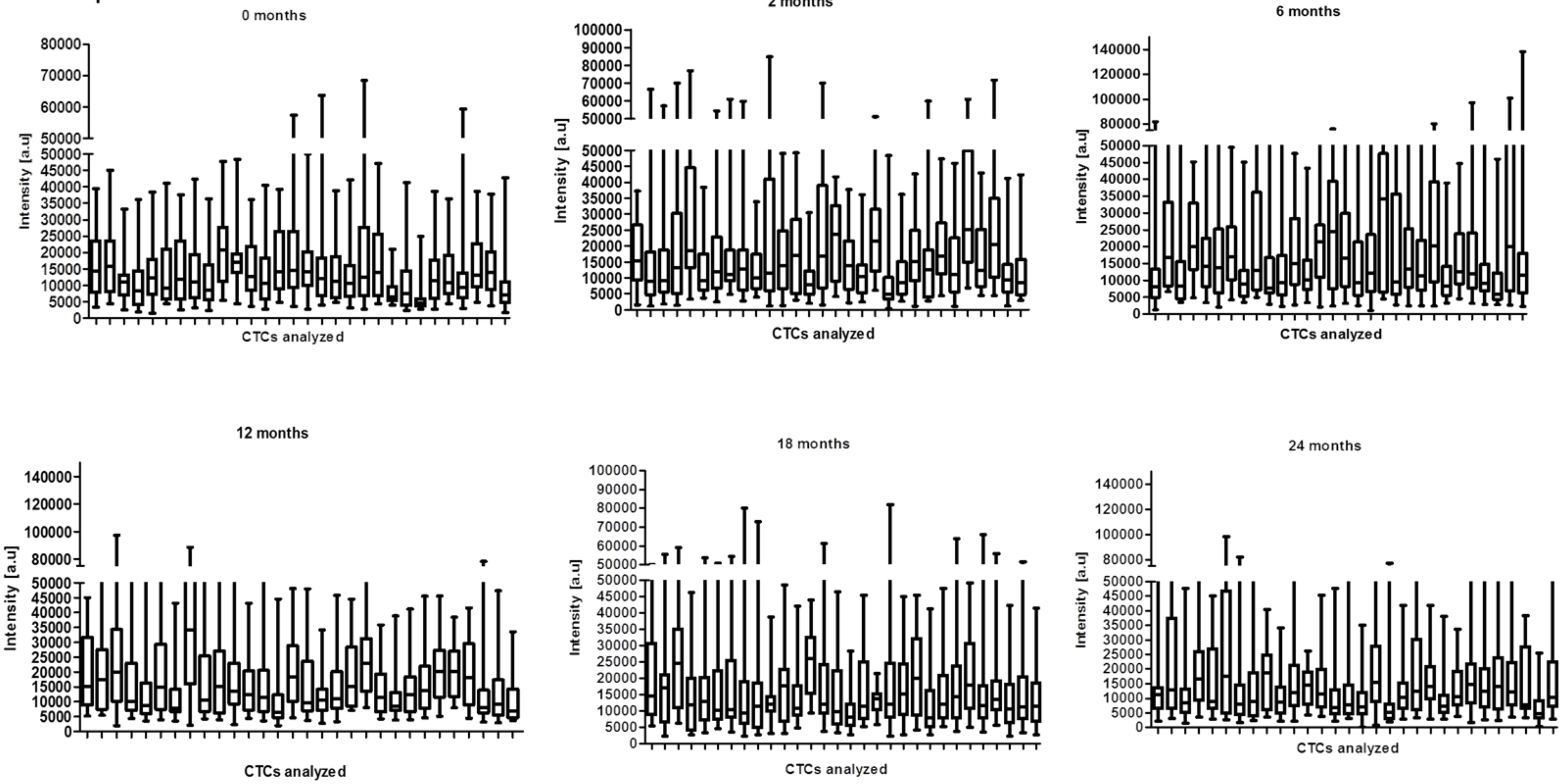

0 months
2 months
6 months
12 months
18 months
24 months
Intensity [a.u]
CTCs analyzed

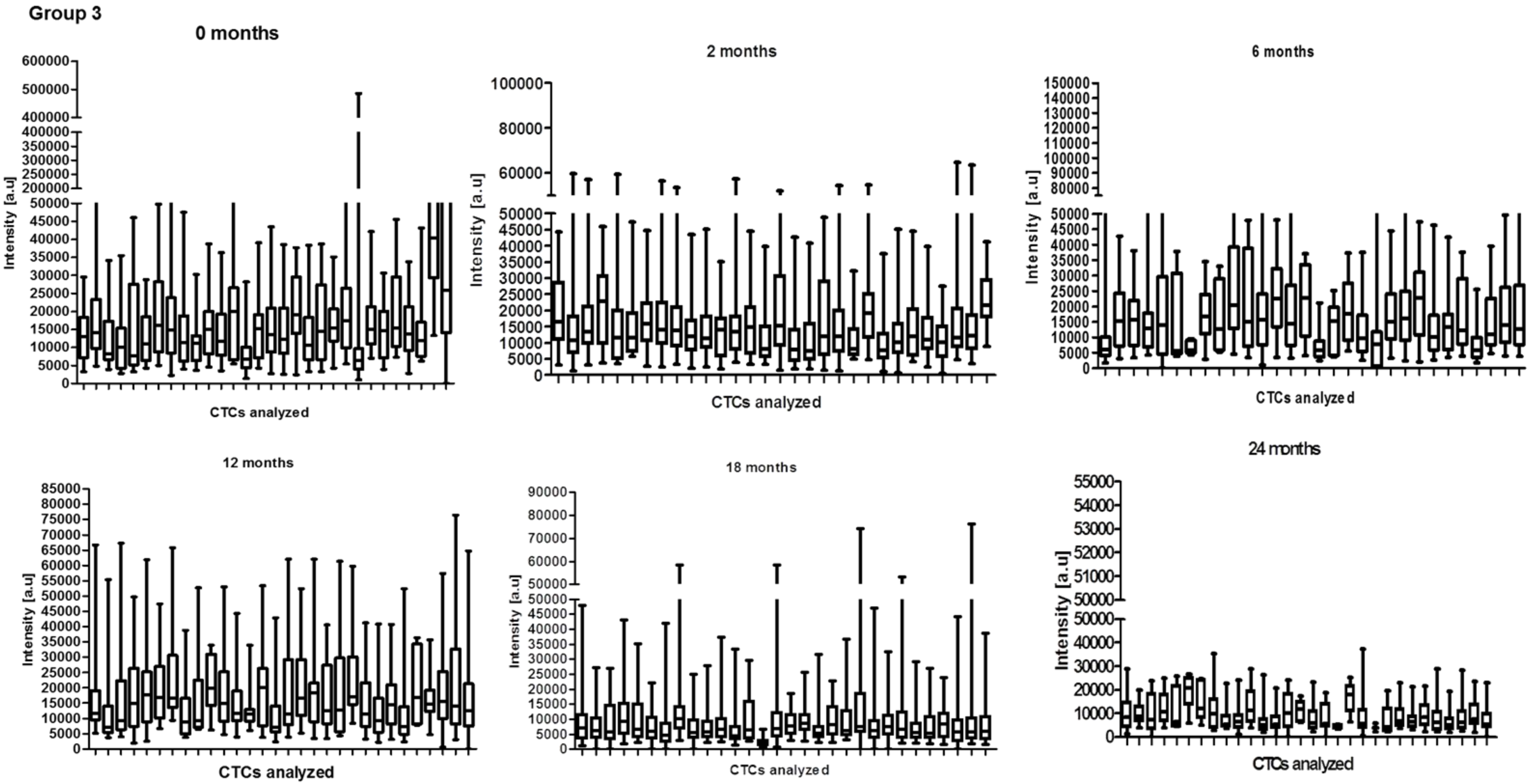
Group 3
0 months
2 months
6 months
12 months
18 months
24 months
Intensity [a.u]
CTCs analyzed

Group 4

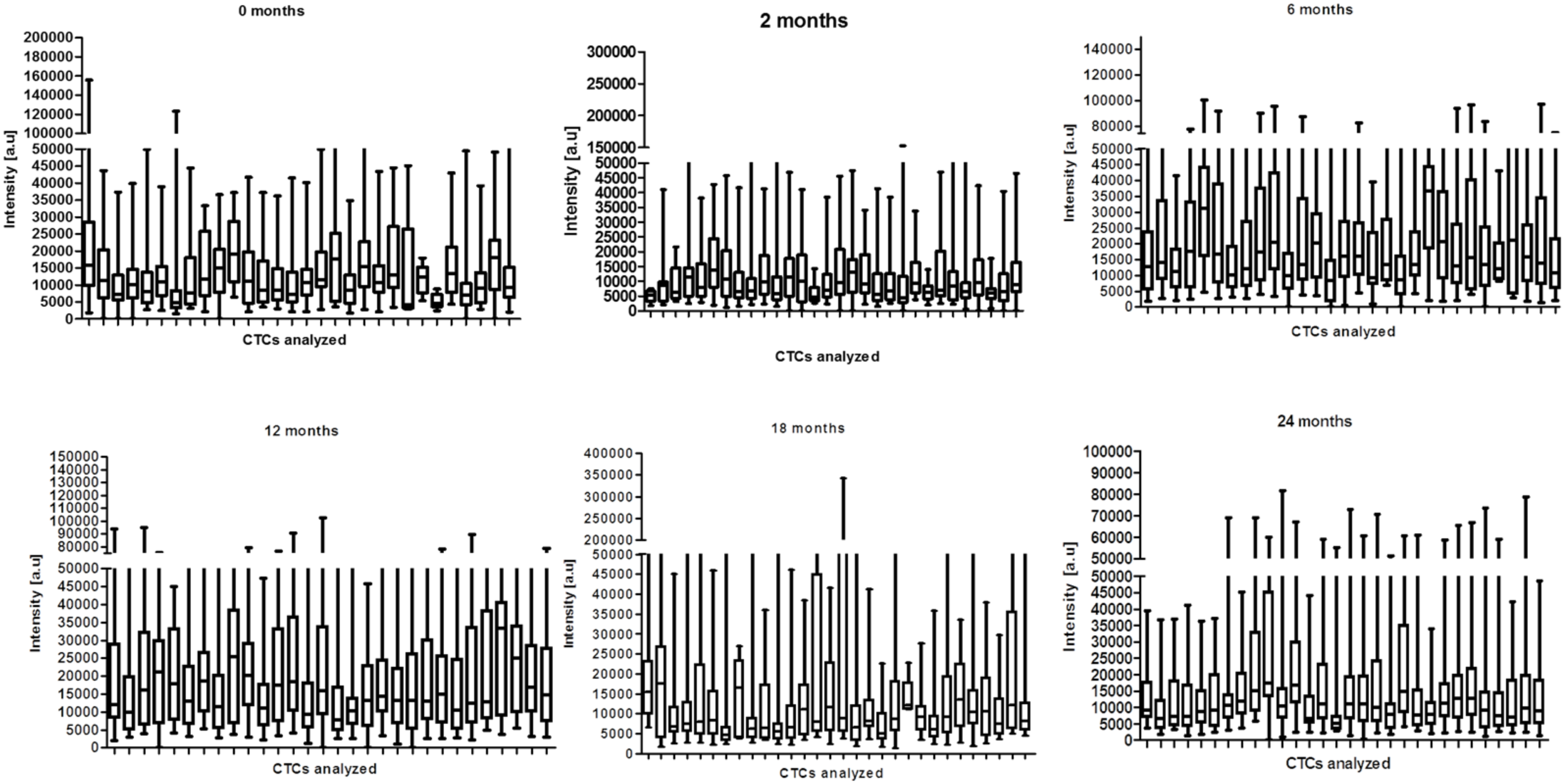
0 months
200000
180000
160000
140000
120000
100000
50000
45000
40000
35000
30000
25000
20000
15000
10000
5000
0
Intensity [a.u]
CTCs analyzed
2 months
300000
250000
200000
150000
50000
45000
40000
35000
30000
25000
20000
15000
10000
5000
0
Intensity [a.u]
CTCs analyzed
6 months
140000
120000
100000
80000
50000
45000
40000
35000
30000
25000
20000
15000
10000
5000
0
Intensity [a.u]
CTCs analyzed
12 months
150000
140000
130000
120000
110000
100000
90000
80000
50000
45000
40000
35000
30000
25000
20000
15000
10000
5000
0
Intensity [a.u]
CTCs analyzed
18 months
400000
350000
300000
250000
200000
50000
45000
40000
35000
30000
25000
20000
15000
10000
5000
0
Intensity [a.u]
CTCs analyzed
24 months
100000
90000
80000
70000
60000
50000
50000
45000
40000
35000
30000
25000
20000
15000
10000
5000
0
Intensity [a.u]
CTCs analyzed

Group 5

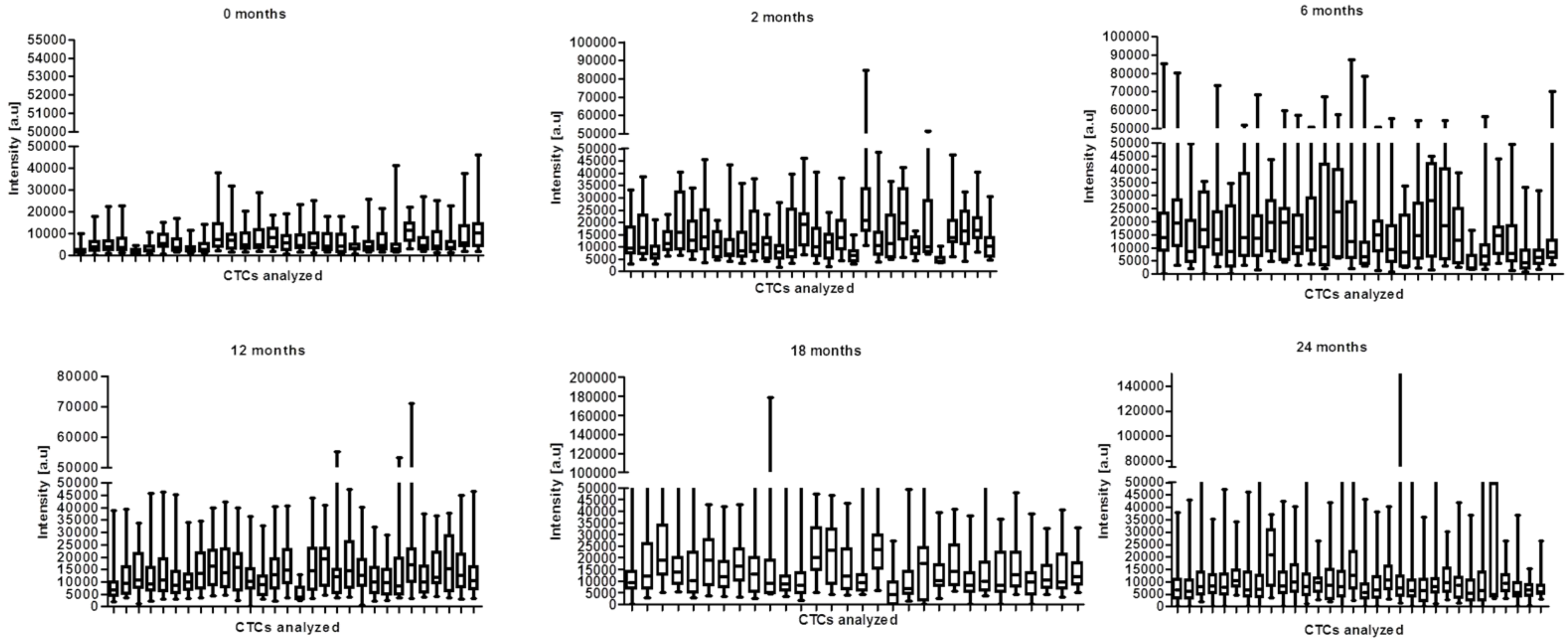

Supplementary Figure 1. Representative bar plots to illustrate inter-sample variability of representative individual samples in the Groups 1, Group 2, Group 3, Group 4, and Group 5.

A

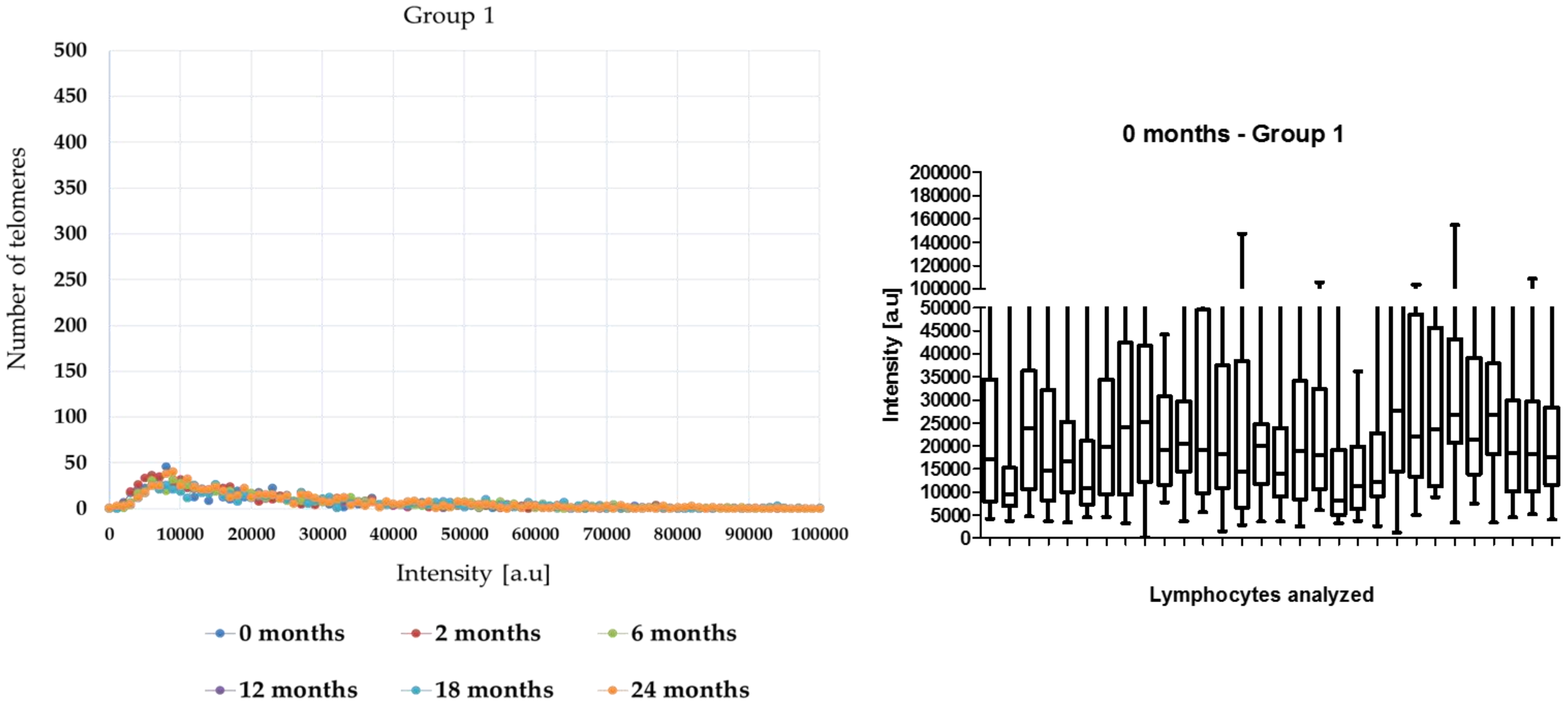
Group 1
Number of telomeres
500
450
400
350
300
250
200
150
100
50
0
0
10000
20000
30000
40000
50000
60000
70000
80000
90000
100000
Intensity [a.u]
0 months
2 months
6 months
12 months
18 months
24 months
0 months - Group 1
Intensity [a.u]
200000
180000
160000
140000
120000
100000
50000
45000
40000
35000
30000
25000
20000
15000
10000
5000
0
Lymphocytes analyzed

B

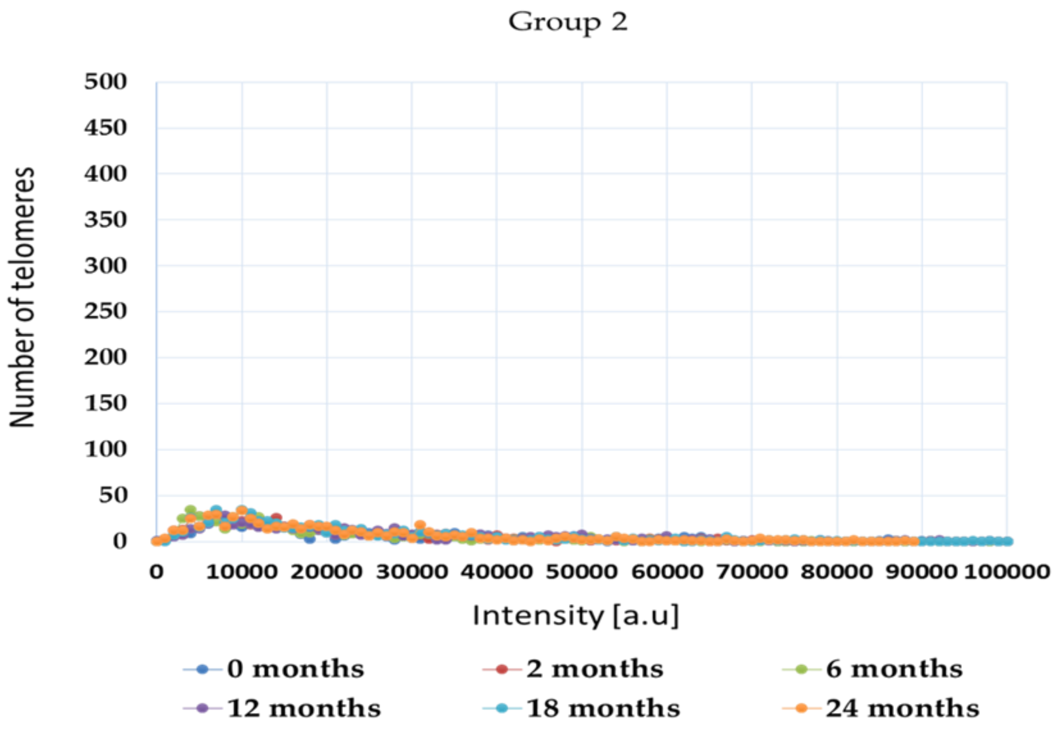
Group 2
Number of telomeres
500
450
400
350
300
250
200
150
100
50
0
0
10000
20000
30000
40000
50000
60000
70000
80000
90000
100000
Intensity [a.u]
0 months
2 months
6 months
12 months
18 months
24 months

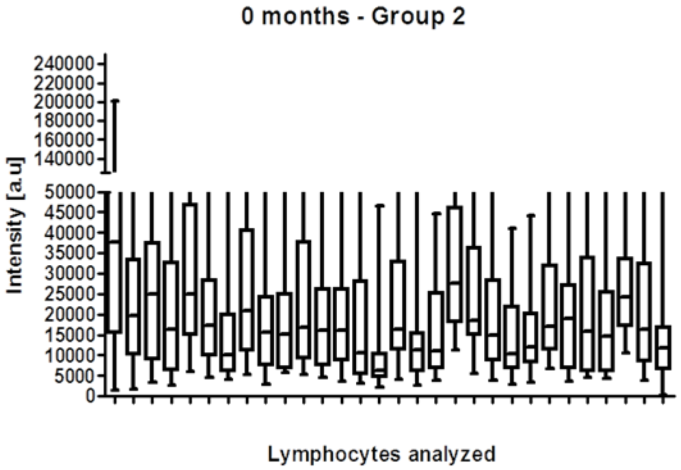
0 months - Group 2
Intensity [a.u]
240000
220000
200000
180000
160000
140000
50000
45000
40000
35000
30000
25000
20000
15000
10000
5000
0
Lymphocytes analyzed

C

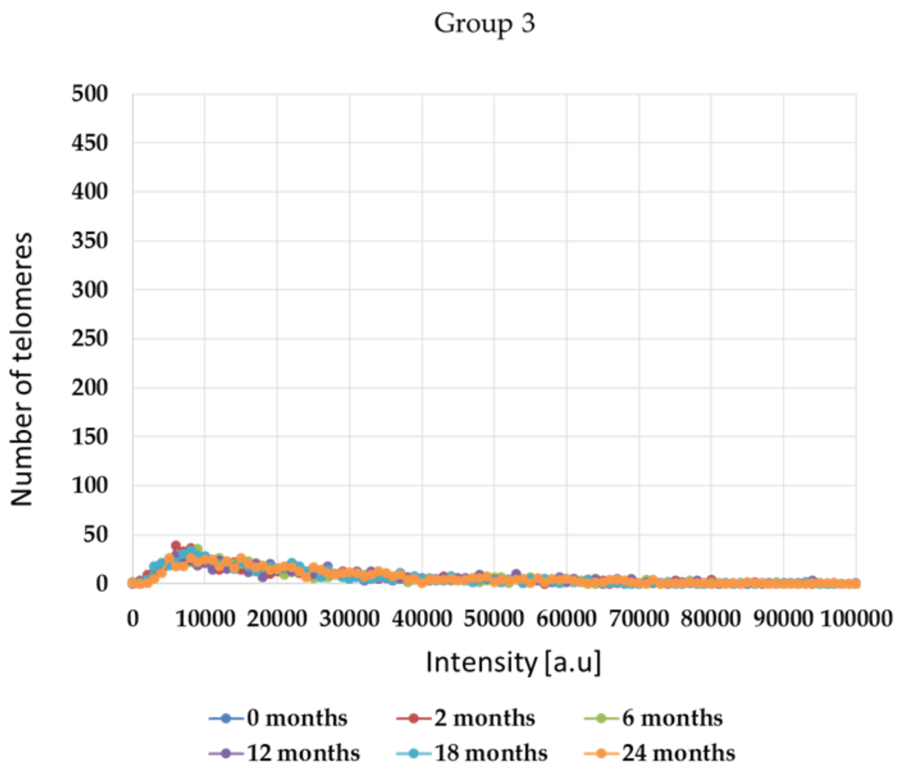
Group 3
Number of telomeres
500
450
400
350
300
250
200
150
100
50
0
0
10000
20000
30000
40000
50000
60000
70000
80000
90000
100000
Intensity [a.u]
0 months
2 months
6 months
12 months
18 months
24 months

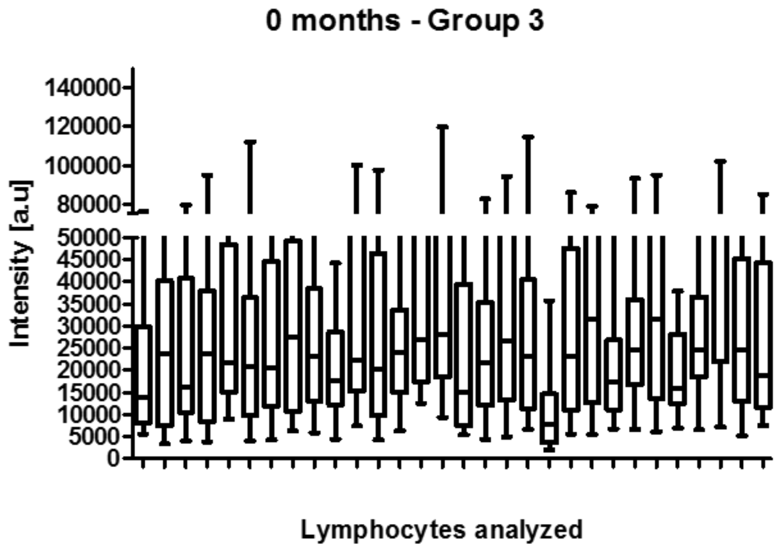
0 months - Group 3
Intensity [a.u]
140000
120000
100000
80000
50000
45000
40000
35000
30000
25000
20000
15000
10000
5000
0
Lymphocytes analyzed

D

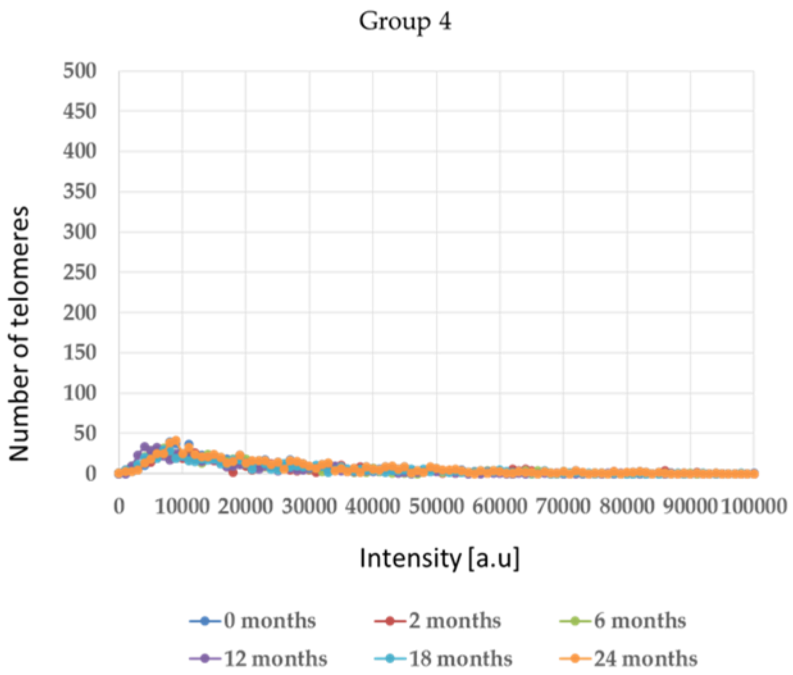
Group 4
500
450
400
350
300
250
200
150
100
50
0
Number of telomeres
0
10000
20000
30000
40000
50000
60000
70000
80000
90000
100000
Intensity [a.u]
0 months
2 months
6 months
12 months
18 months
24 months

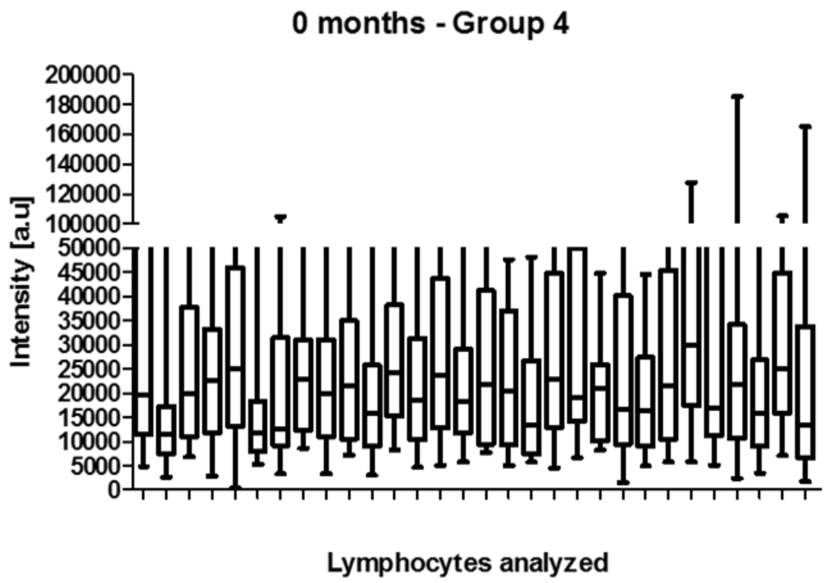
0 months - Group 4
200000
180000
160000
140000
120000
100000
50000
45000
40000
35000
30000
25000
20000
15000
10000
5000
0
Intensity [a.u]
Lymphocytes analyzed

E

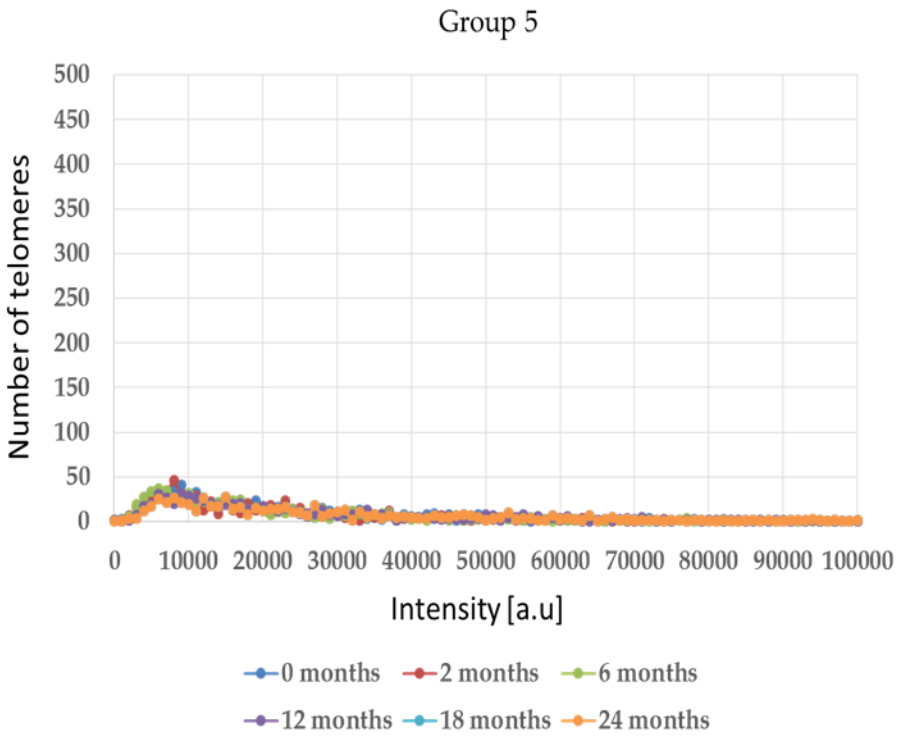

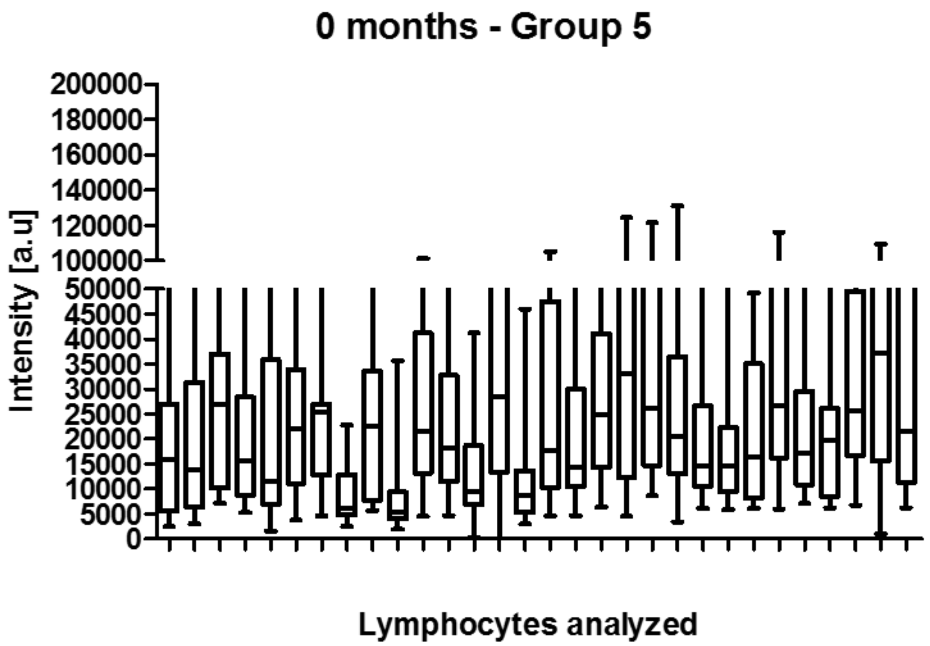

Supplementary Figure 2. Representative examples of the lymphocytes (internal control) dynamics of telomere length profiles over time for patients assigned to Group 1 (A), Group 2 (B), Group 3 (C), Group 4 (D), and Group 5 (E). In each graph, the telomere length is shown in arbitrary units of fluorescence (AU). Baseline profile (+0m, untreated) and other time point (2, 6, 12, 18, 24 months) are demarked with colors. B) bars plot to illustrate inter-sample variability of representative individual samples in the groups (for simplicity just the 0 month – untreated time point was represented).

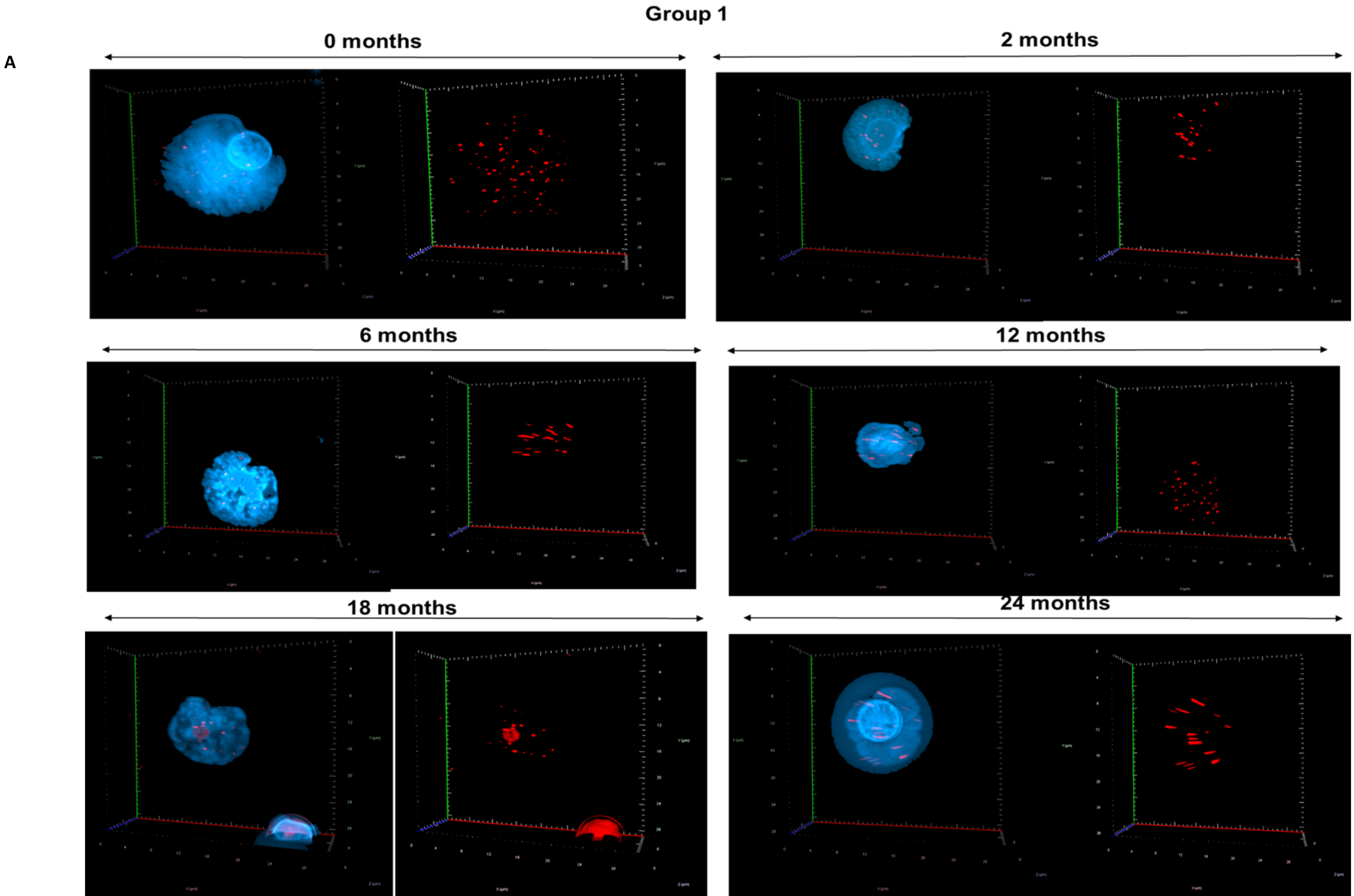
Group 1
A
0 months
2 months
6 months
12 months
18 months
24 months

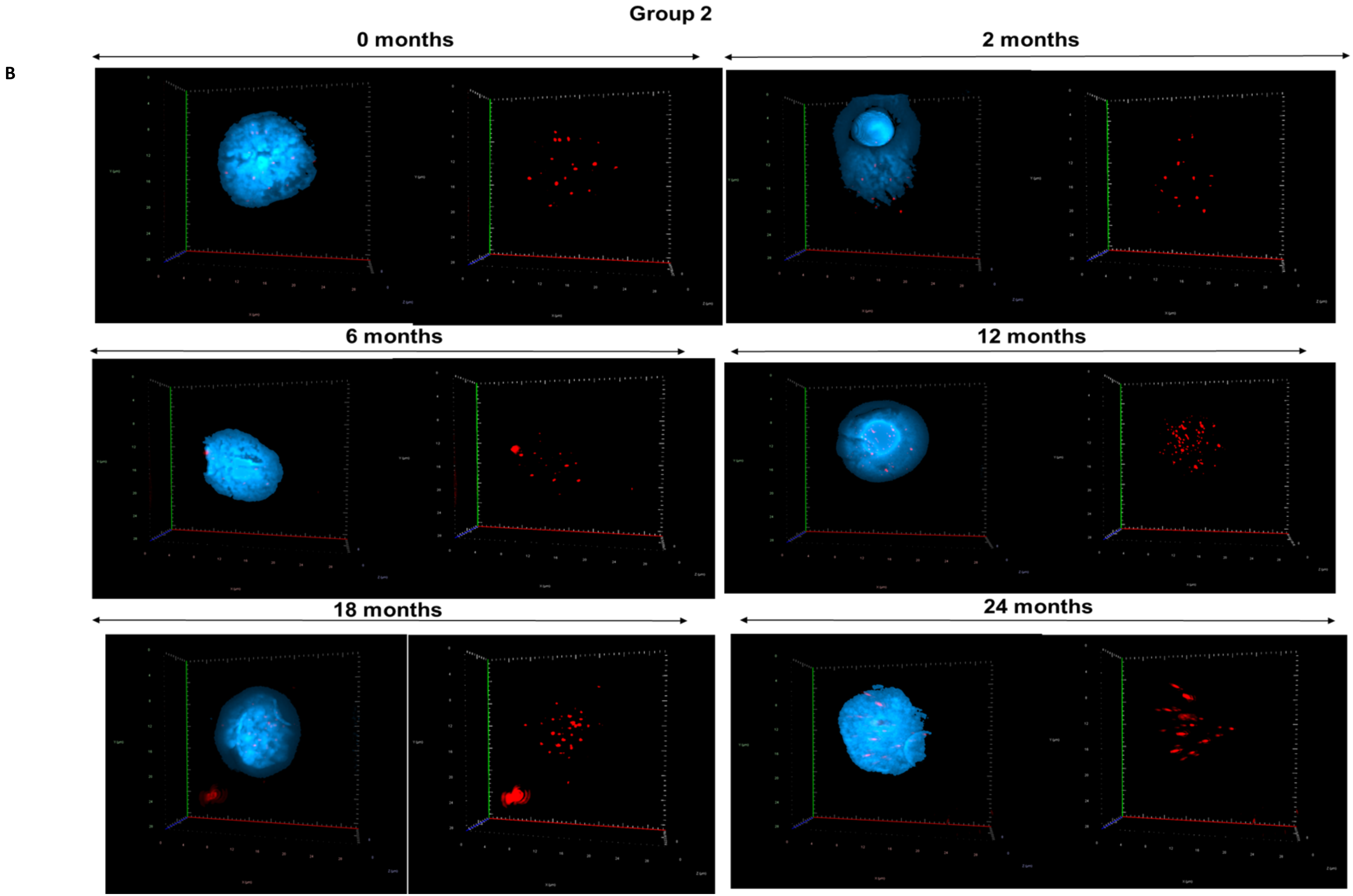
Group 2
B
0 months
2 months
6 months
12 months
18 months
24 months

C

Group 3

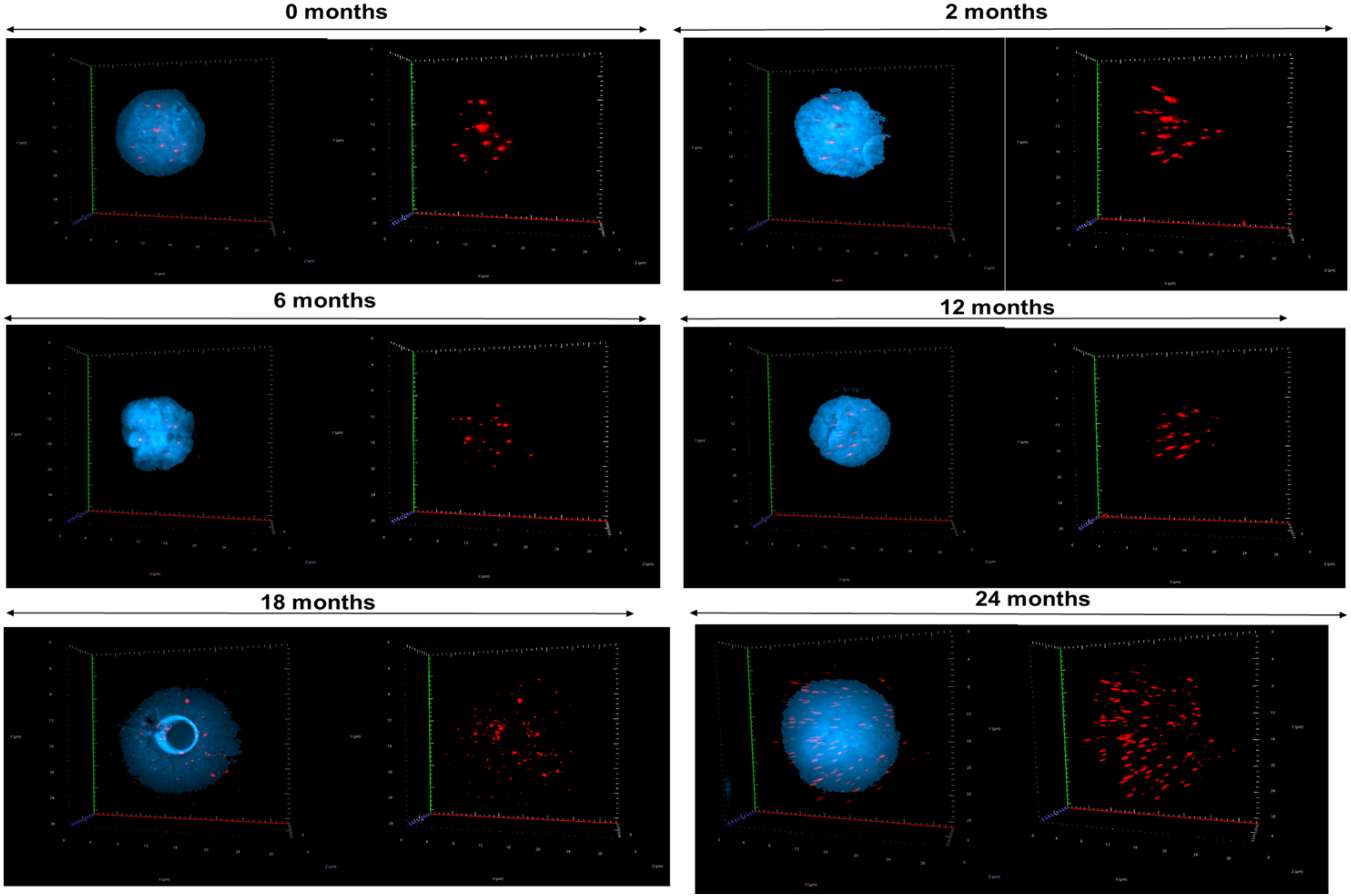

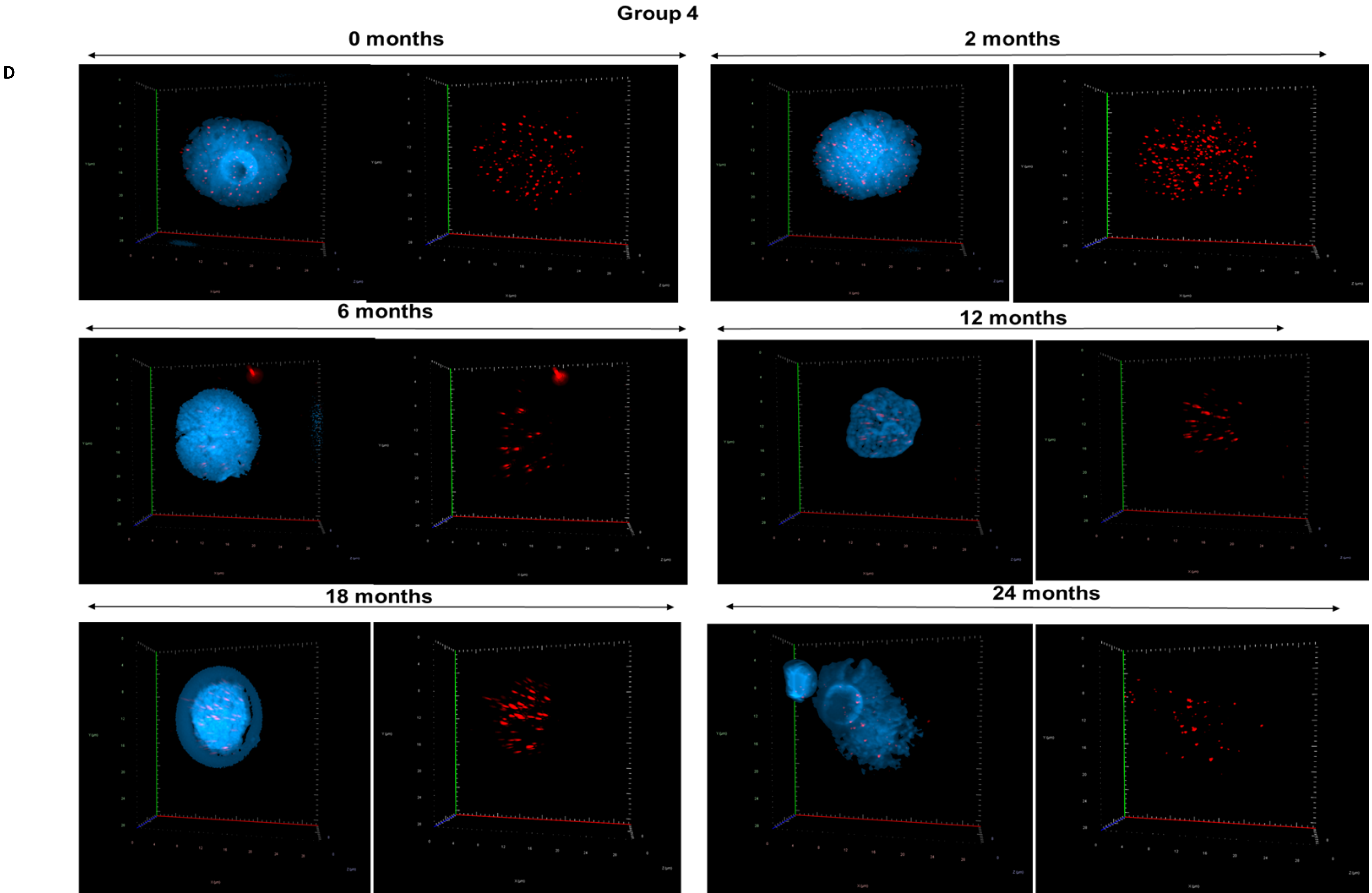
Group 4
D
0 months
2 months
6 months
12 months
18 months
24 months

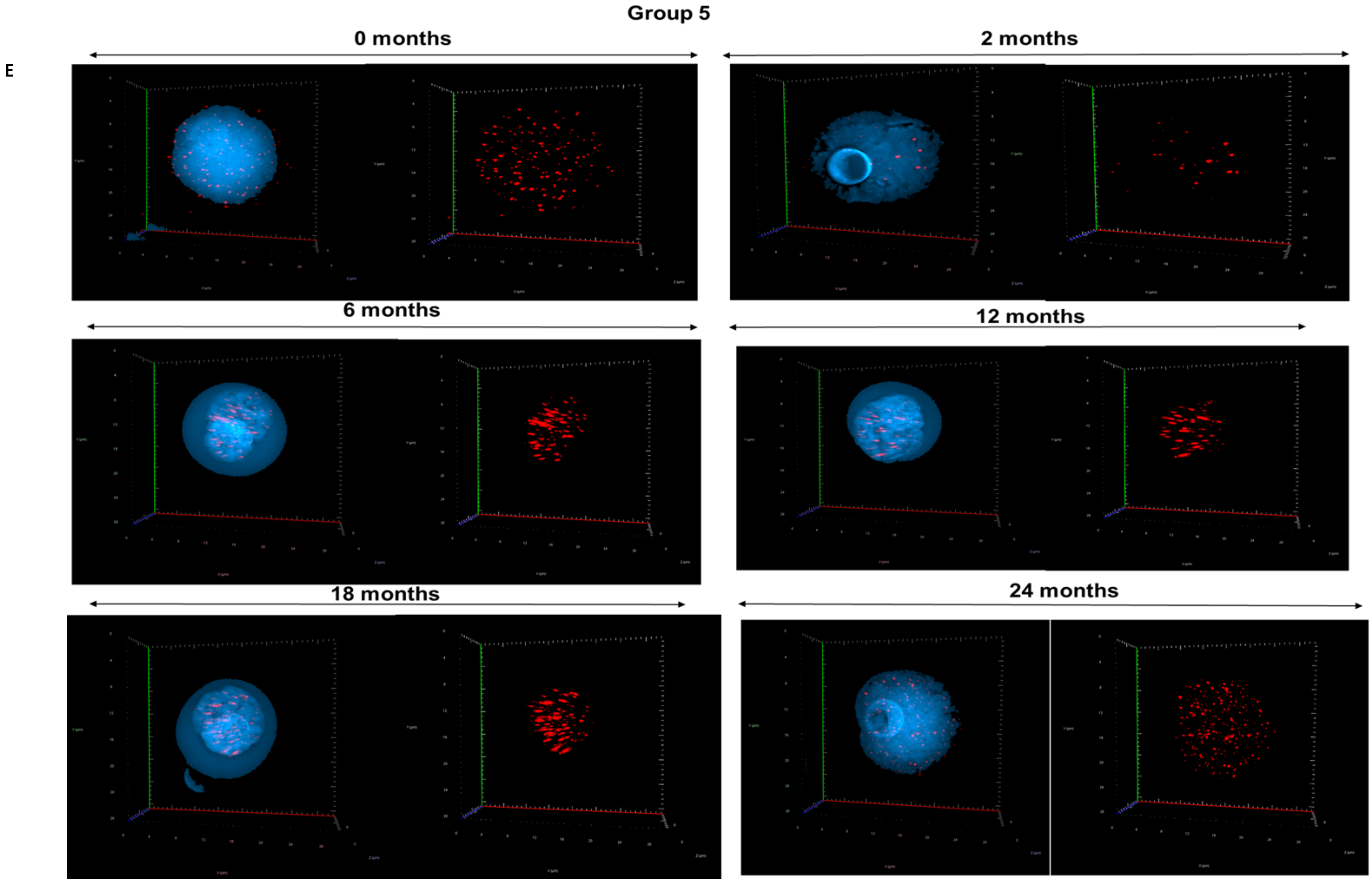
Group 5
E
0 months
2 months
6 months
12 months
18 months
24 months

Supplementary Figure 3. Example of a circulating tumor cell from a high-risk localized prostate cancer patient captured on top of a filter pore for each time point. Three-dimensional representation of a CTC with the telomeres labeled with telomere-specific Cy3-labeled probe (red) and Merge between telomeres; and the counterstained DAPI (blue).
